# A Genome-Wide Investigation of Effects of Aberrant DNA Methylation on the Usage of Alternative Promoters in Hepatocellular Carcinoma

**DOI:** 10.3389/fonc.2021.780266

**Published:** 2022-01-17

**Authors:** Yuting Dong, Xiaozhao Liu, Bijun Jiang, Siting Wei, Bangde Xiang, Ruichu Liao, Qiuyan Wang, Ximiao He

**Affiliations:** ^1^Department of Physiology, School of Basic Medical Science, Huazhong University of Science and Technology, Wuhan, China; ^2^Center for Genomics and Proteomics Research, School of Basic Medicine, Tongji Medical College, Huazhong University of Science and Technology, Wuhan, China; ^3^Hubei Key Laboratory of Drug Target Research and Pharmacodynamic Evaluation, Huazhong University of Science and Technology, Wuhan, China; ^4^Department of Hepatobiliary Surgery, Guangxi Medical University Cancer Hospital, Nanning, China; ^5^Center for Genomic and Personalized Medicine, Guangxi Medical University, Nanning, China; ^6^Guangxi Key Laboratory for Genomic and Personalized Medicine, Guangxi, Collaborative Innovation Center for Genomic and Personalized Medicine, Nanning, China

**Keywords:** hepatocellular carcinoma, alternative promoters, DNA methylation, diagnostic model, survival prediction

## Abstract

**Background:**

The alternative usage of promoters provides a way to regulate gene expression, has a significant influence on the transcriptome, and contributes to the cellular transformation of cancer. However, the function of alternative promoters (APs) in hepatocellular carcinoma (HCC) has not been systematically studied yet. In addition, the potential mechanism of regulation to the usage of APs remains unclear. DNA methylation, one of the most aberrant epigenetic modifications in cancers, is known to regulate transcriptional activity. Whether DNA methylation regulates the usage of APs needs to be explored. Here, we aim to investigate the effects of DNA methylation on usage of APs in HCC.

**Methods:**

Promoter activities were calculated based on RNA-seq data. Functional enrichment analysis was implemented to conduct GO terms. Correlation tests were used to detect the correlation between promoter activity and methylation status. The LASSO regression model was used to generate a diagnostic model. Kaplan–Meier analysis was used to compare the overall survival between high and low methylation groups. RNA-seq and whole-genome bisulfite sequencing (WGBS) in HCC samples were performed to validate the correlation of promoter activity and methylation.

**Results:**

We identified 855 APs in total, which could be well used to distinguish cancer from normal samples. The correlation of promoter activity and DNA methylation in APs was observed, and the APs with negative correlation were defined as methylation-regulated APs (mrAPs). Six mrAPs were identified to generate a diagnostic model with good performance (AUC = 0.97). Notably, the majority of mrAPs had CpG sites that could be used to predict clinical outcomes by methylation status. Finally, we verified 85.6% of promoter activity variation and 92.3% of methylation changes in our paired RNA-seq and WGBS samples, respectively. The negative correlation between promoter activity and methylation status was further confirmed in our HCC samples.

**Conclusion:**

The aberrant methylation status plays a critical role in the precision usage of APs in HCC, which sheds light on the mechanism of cancer development and provides a new insight into cancer screening and treatment.

## Introduction

Liver cancer is the sixth most prevalent cancer and fourth most lethal malignancy globally (1). Hepatocellular carcinoma (HCC) is the dominant tissue subtype of aggressive primary liver cancer, accounting for a great majority of the diagnoses and deaths (2). HCC prognosis is poor worldwide, with the 5-year survival rate ranging from 5% to 30% (3). The main treatment options for patients with HCC include vascular intervention, surgical resection, radiofrequency ablation, or liver transplantation. Most patients have reached the advanced stage of HCC when they are first diagnosed, and only about 20%–30% of patients are eligible for effective treatment (4). Early detection with surveillance is the most effective way to reduce the mortality of HCC (5). Further study on the pathogenesis of HCC is of great significance to the diagnosis and prognosis of tumors.

Promoters are the key element in regulating gene expression. In human genomes, most protein-coding genes are co-regulated by numerous promoters (6). The differential usage of promoters has been reported to be highly correlated with disease. For example, the dominance of c-MYC, which is silent in normal tissue, is abnormally activated in Burkitt lymphoma cells as a result of aberrant alternative promoter (AP) usage at the *MYC* gene locus (7). Another well-studied AP example is *RASSF1*, which encodes different subtypes RASSF1A and RASSF1C. The former acted as a tumor suppressor gene and the latter had carcinogenic activity (8). These studies of differential promoter usage usually focused on single genes. With the development of sequencing technology, approaches of detecting genome-wide promoter activities were available, including H3K4me3 ChIP-seq (9), Cap Analysis of Gene Expression (CAGE) (10), and short-read (11) and long-read (12) sequencing of RNAs. It is worth noting that the approach to predict promoter activity based on RNA-seq of short reads has a good consistency with previous methods (11). Previous studies have shown that increasing AP repertories is accompanied by elevated differential expression and disease susceptibility (13). In addition, tissue-specific promoter activity could be used to distinguish different cancer subtypes (12).

As one of the most essential epigenetic modifications, DNA methylation is involved in oncogenesis (14, 15). In various cancers, gene expression could be silenced by hypermethylation of promoter regions by the interfering transcription factors binding or recruitment of transcriptional repressors (16–18), while the overexpression of oncogenic drivers (19) or instability of chromosomes (20) could be associated with hypomethylated regions. Therefore, DNA methylation detection may be helpful to elucidate molecular mechanisms of HCC development (21). Furthermore, changes in DNA methylation could be used as promising targets for diagnosis or prognosis biomarkers in HCC (22, 23). For example, methylation of the *GSTP1* promoter has been reported as a diagnostic marker and indicates poor outcomes (24). Due to the stability and non-invasive detectability in blood, circulating tumor DNA (ctDNA) methylation markers have also been reported for HCC diagnosis in several studies (25–27).

In HCC, differential usage of promoters has not been systematically studied, and whether DNA methylation regulates the usage of APs in HCC remains unclear. Here, we firstly systematically analyzed the promoter activities and identified the APs in HCC, and the results indicated that APs could distinguish cancer from normal cells. Then, we correlated the promoter activity of APs with DNA methylation, and the results suggested that AP activity could be regulated by the methylation changes. Furthermore, a diagnostic model by methylation-related APs was generated and the methylation of APs could also be used as prognostic markers, which indicated that AP-related methylation has the potential for molecular diagnoses and prognosis prediction of HCC. Finally, RNA-seq and WGBS were performed to verify the correlation of the promoter activity and methylation status of APs in HCC.

## Materials and Methods

### Data Collection

The RNA-seq raw data and Infinium Human Methylation 450 K Bead Chip (Illumina 450 K array) matrix data of liver tissues (19 HCC and 19 paired adjacent normal samples) were downloaded from the Gene Expression Omnibus (GEO) cohort (https://www.ncbi.nlm.nih.gov/geoprofiles) GSE77276 (28). Two other independent cohorts of HCC RNA-seq data were downloaded from GSE55758 (8 HCC and 8 paired adjacent normal samples) (29) and GSE105130 (25 HCC and 25 paired adjacent normal samples) (30). In addition, an independent cohort of two paired HCC and normal liver samples with RNA-seq raw data and whole-genome bisulfite sequencing (WGBS) methylation data was downloaded from GSE70091 (31). Liver cancer Illumina 450 K array and related clinical details of GDC TCGA Liver Cancer (LIHC) were downloaded from the UCSC Xena database (32) (https://xenabrowser.net/datapages/). The ONGene and TSGene lists were downloaded from ONGene (33) (http://ongene.bioinfo-minzhao.org/) and TSGene (34) (http://bioinfo.mc.vanderbilt.edu/TSGene/).

### Validation Samples Collection

The fresh-frozen tissue specimens were collected from two HCC patients from Guangxi Medical University Cancer Hospital for validation. For each patient, tumor tissues and adjacent normal liver tissues were collected through surgery. Each fresh tissue was aliquoted into three pieces and separately stored at liquid nitrogen using a cryopreservation tube until DNA and RNA extraction. All samples were sequenced by both RNA-seq and Whole Genome Bisulfite Sequencing (WGBS).

### RNA-seq and Data Processing

The total RNA was extracted with HiPure Universa miRNA Kit (Magen) from two pairs of fresh frozen tissue samples and quality was confirmed by Nanodrop measurement of OD 260/280 and 260/230 ratios. The material for library construction was 1 μg per sample. Sequencing libraries were constructed following the Illumina TruSeq Stranded protocol. Total RNA Gold kit with Ribo-Zero Gold (Illumina, USA) was used following the manufacturer’s recommendations. Sequencing (2×150 paired-end reads) was performed at Mingma Technologies Co., Ltd in Shanghai.

FASTQ format data were assessed using FastQC (v0.11.9) and then fastp (v0.20.1) (35) was used to remove the bases with an average quality value less than 20 and to cut the reads of adapters. Clean reads were mapped to the human reference genome (Gencode v19) by STAR (2.7.5b) (36). The gene and transcript isoform expression was quantified using RSEM (v1.3.1) (37). Bedtools (v2.29.2) (38) was used to transform the bam files to bw format for UCSC genome browser viewing.

### Whole-Genome Bisulfite Sequencing

HiPure Tissue DNA Mini Kit (Magen) was used for tissue genomic DNA extraction. After quantification by Qubit fluorometer, 1% unmethylated Lambda DNA was added to 200 ng of gDNA, and then randomly fragmented to 300-bps insert size with Covaris LE220. After end repair and adenylation, methylated adapters were ligated to the fragmented DNA. Bisulfite treatment was performed according to the EZ DNA Methylation-Gold kit (Zymo Research) instruction manual. KAPA HiFi HotStart Uracil + ReadyMix (2×) was used to amplify and purify the DNA fragments. Next, the Qubit Fluorometer dsDNA HS Assay (Thermo Fisher Scientific) and Agilent BioAnalyzer (Agilent) were used to measure and analyze the size distribution of the sequencing library; 2×150 paired-end reads sequencing is performed using an Illumina NovaSeq6000 following Illumina-provided protocols at Mingma Technologies Co., Ltd.

### WGBS Data Preprocessing

Standard WGBS data analysis pipeline was followed. The raw FASTQ data were firstly trimmed, adapters were removed using TrimGalore (v0.4.3), and approximately 42 Gbps of data were reserved. Clean reads were next aligned with the human reference genome (hg19) using BSMAP (v2.89) (39). Mapped BAM files were then sorted and PCR deduplicates were removed through SAMtools (v. 1.3.1) and Picard Tools (v.1.92). Finally, MOABS (v. 1.3.4) (40) was used to calculate the methylation ratio per CpG. In promoter regions (−2 kb to 2 kb around TSS), methylation profile was smoothed by gam (Generalized Additive Models) or 50-bps sliding windows with 25-bps steps.

### Methylation Analysis of 450K Methylation Array

In the 450K methylation array matrix, the delta mean beta (*β*) was calculated by *β* (mean tumor) − *β* (mean normal). A positive delta *β* value indicated relative hypermethylation in the tumor while a negative delta *β* value exhibited relative hypomethylation. The paired Student’s *t*-test was used for statistics. Methylation profile in promoter regions (−2 kb to 2 kb around TSS) smoothed using the same method as above.

### Promoter Identification and Activity Estimation

The R package “proActiv” (v.0.99.0) was used to identify possible promoters and calculate the promoter activity. GTF files (Gencode v19) and STAR junction files were used as input. Promoter activity was obtained by removing single-exon transcripts/promoters and eliminating promoter counts that are NA and zero both in tumor and normal samples. When identifying the differentially regulated promoters (DRPs), the internal promoter activity was also considered.

### Differential Analysis of Gene Expression and Promoter Activity

Differential analysis of gene expression was performed by the R package “DESeq2” (v1.28.0) [*p-*value < 0.05 and |log_2_(Fold Change)| > 1]. The degree of promoter change is calculated by log_2_ [promoter activity (mean tumor)/promoter activity (mean normal)]. For each promoter, the one-way analysis of variance (ANOVA) was used to assess the significance of absolute and relative promoter activity variance between tumor and normal samples. The promoters with an activity change level of |Fold Change| > 1.2 and *p-*value < 0.05 were considered significant DRPs.

### Identification of Alternative Promoters

We identified APs by screening both gene expression and promoter activity. The criteria were as follows: (1) *p-*value ≥ 0.05 and |Fold Change| < 2 of gene expression; (2) mean absolute promoter activity > 0.25 in HCC and normal group; (3) both absolute and relative promoter activity were significantly changed (*p-*value < 0.05); (4) |Fold Change| > 1.2 of absolute promoter activity.

### Dimensionality Reduction and Clustering

Gene expression and promoter activity were subjected to dimensionality reduction using the principal component analysis (PCA) and t-Distributed Stochastic Neighbor Embedding (t-SNE) through R packages “stats” and “Rtsne”.

### Functional Enrichment Analysis

To identify the possible functions and pathways of hub genes, gene set enrichment analysis was implemented to conduct GO terms through Metascape (41) online (http://metascape.org/). *p-*value < 0.01 was used as the cutoff criteria.

### Correlation Analysis

In the correlation analysis of CpG methylation and gene expression or promoter activity, the representative CpG sites of 450K were selected as follows: (1) For each CpG site upstream and downstream ±1kb of TSS, Pearson correlation test between methylation and gene expression (or promoter activity) was calculated. (2) The CpG sites with a minimum *p-*value of Pearson correlation test were selected to represent the methylation level of gene or promoter. Gene expression change [log_2_(Fold Change)] was obtained from Deseq2, and promoter activity change was normalized by log_2_(Fold Change) of promoter activity. For the WGBS methylation data of validation part, both representative CpG sites and mean methylation levels of ±1kb of TSS were calculated for promoter methylation, and Pearson correlation test was used for all correlation analysis.

### LASSO Regression Analysis

The LASSO regression analysis of binary data was applied to construct a diagnosis model by the R package “glmnet”. The penalty parameter (λ) of this diagnosis model was confirmed through 10-fold cross-validation. The risk score was calculated as follows: model Score = ∑ (promoter activity × regression coefficient). The GSE55758 and GSE105130 datasets were both used for further cross-verification. Receiver operating characteristic (ROC) curves were used to visualize the reliability of the diagnostic model, and the area under the curve (AUC) was also calculated.

### Survival Analysis

Kaplan–Meier analysis in 10 years was performed in the R software “survival” package. All samples were classified into two groups according to the best-performed cutoff methylation *β* value using the “surv-cutpoint” function. *p-*value < 0.05 was considered statistically significant.

### Statistical Analysis

R version 4.0.2 was used for all statistical analysis and visualization. Statistical analysis was performed through R base package stats (v4.0.2). All figures were generated using ggplot2 (v3.1.0) and ggpubr (v0.2). Significance levels were defined as follows: ns: *p* > 0.05, *p < 0.05, **p < 0.01, ***p < 0.001, and ****p < 0.0001 in boxplot.

## Results

### The Landscape of Promoter Activities in HCC

**Figure 1:**
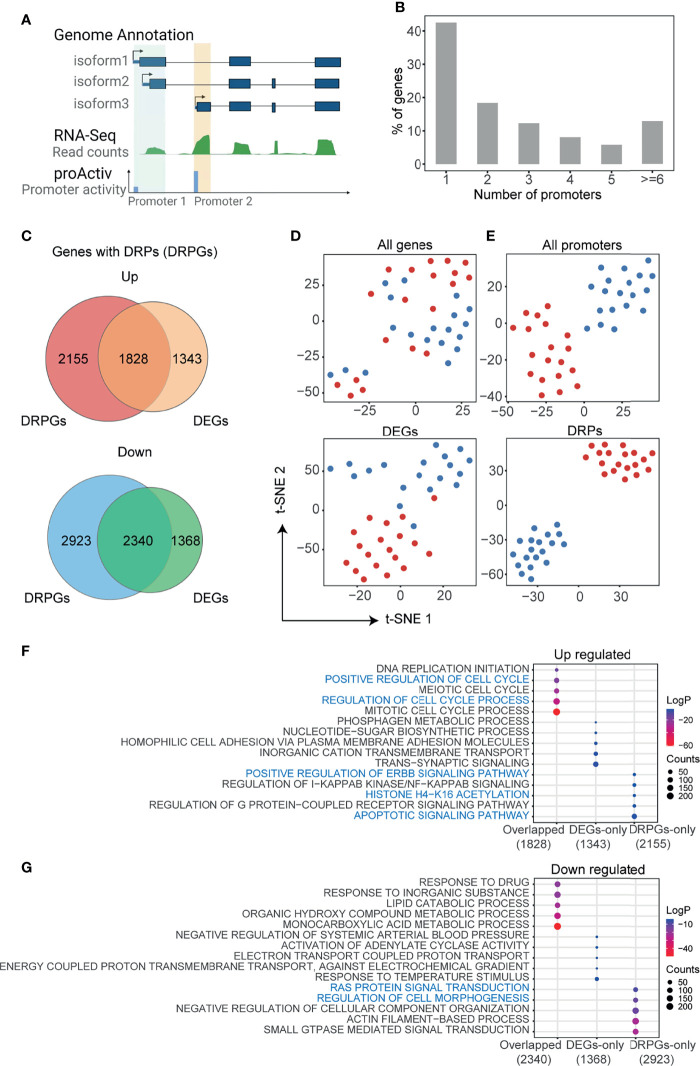
Analysis of promoter activities in HCC. **(A)** Schematic representation of promoter activities of different transcript isoforms. Transcripts with the same or nearby transcript start site are grouped into the identical promoter. Promoter activity is defined as the total unique junction reads spanning at each promoter (see also *Materials and Methods*). The green track represents gene expression of tissue normalized by reads counts, blue track represents the activity of each promoter. **(B)** The number of promoters with activities in HCC per gene, a total of 25,085 genes with 70,736 promoters included. **(C)** Venn diagrams showing the overlap of differentially expressed genes (DEGs) and genes with differentially regulated promoters (DRPGs) for upregulated (top) and downregulated (bottom) ones. **(D, E)** t-distributed stochastic neighbor embedding (t-SNE) clustering the sequenced samples by FPKM for all genes or DEGs **(D)** and by promoter activities for all promoters or DRPs **(E)**. Samples were colored by sample types (dark red: HCC; dark blue: adjacent normal tissue). **(F, G)** Bubble plots showing the enriched biological processes by gene ontology (GO) analysis of gene groups in **(C)**. The bubble color represents the log_2_ (*p*-value) while the bubble size represents enriched gene counts.

In mammal genomes, most genes are co-regulated by multiple promoters. As shown in **Figure 1A**, the demo gene has three isoforms but two promoters, because two isoforms (e.g., isofrom1 and isoform2) with the same or nearby transcript start sites (TSS) could be regulated by the identical promoter. To detect the promoter activities in HCC, we analyzed the RNA-seq data of paired HCC and adjusted normal tissues that were downloaded from GEO (GSE77276) (28). RNA-seq reads mapped to the first exons were integrated and normalized to measure the promoter activities by the R package “proActiv” (11). In total, we obtained the activity status of 113,076 possible promoters from the human reference genome, and 70,736 promoter activities of 25,085 genes were obtained from liver tissue. Approximately 57.4% (14,411/25,085) of genes had two or more different promoters (**Figure 1B** and **Supplementary Figure 1A**). Then, we compared the differences in gene expression and promoter activity between tumor and normal tissues, respectively (**Supplementary Figures 1B, C**). We identified 6,879 differentially expressed genes (DEGs) and 8,976 genes with 16,049 DRPs. The upregulated DEGs and genes with DRPs (DRPGs) were partially overlapped, and so were the downregulated ones (**Figure 1C**). Using t-distributed stochastic neighbor embedding (t-SNE), it was hard to distinguish the tumor from normal samples by expression of either all genes or DEGs (**Figure 1D**), whereas distinguishment was successfully achieved by the activities of either all promoters or DRPs (**Figure 1E**). A similar result was obtained through principal component analysis (PCA; **Supplementary Figure 1D**). Those results indicated that promoter activity exhibited a more obvious effect than gene expression in revealing the differences between tumor and normal samples.

Furthermore, we performed functional enrichment analysis on DEG and DRPG overlapped genes, genes unique to DEGs (DEGs-only), and DRPG (DRPGs-only) (**Figures 1F, G**). We noticed that overlapped upregulated genes were associated with proliferation-related ontologies, such as positive regulation of cell cycle and DNA replication. In addition, some cancer-related ontologies, such as regulation of apoptotic signaling pathway, histone acetylation, and positive regulation of ERBB signaling pathway, were enriched in DRPGs-only (**Figure 1F**). In downregulated genes, only DRPGs can be specifically enriched to the regulation of cell morphogenesis or Ras protein signal transduction (**Figure 1G**). Taken together, those results supported that there was general diversity in promoter activities between HCC and normal tissues. Compared with traditional gene expression analysis, the promoter activity analysis was more effective and more accurate in distinguishing HCC from normal tissues, which could provide more clues to investigate the potential mechanism of tumorigenesis and development.

### Identification of Alternative Promoters in HCC

**Figure 2:**
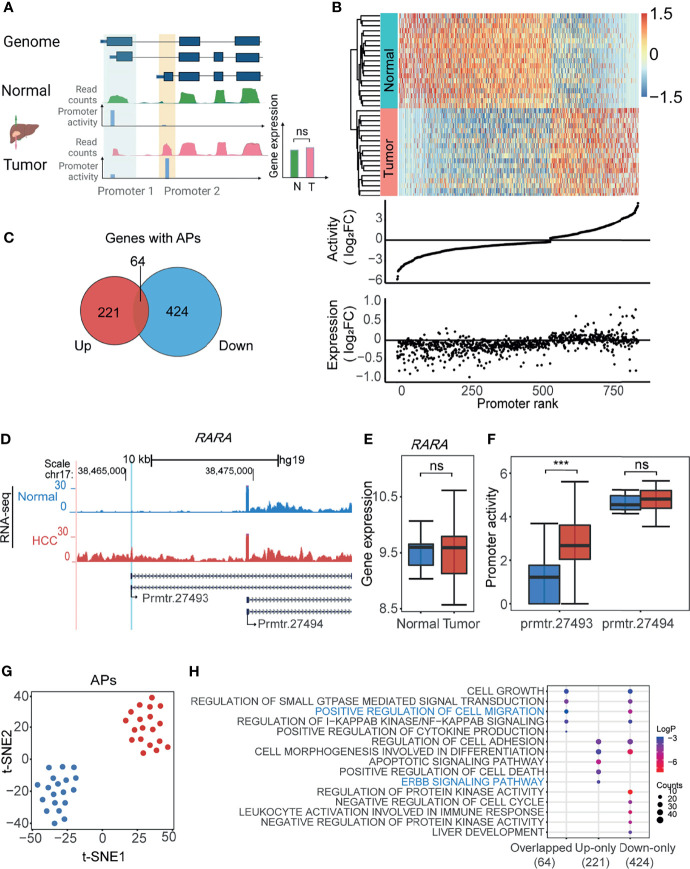
Identification of alternative promoters (APs) in HCC. **(A)** The schematic illustration of the approach to identify APs in HCC. The promoters with differential activities (tumor vs. normal) but without differential expression were defined as APs (see also *Materials and Methods*). Green track and red track represent gene expression of normal and tumor tissue normalized by reads counts; blue tracks represent the activity of each promoter. **(B)** Heatmap showing the normalized promoter activities of APs for HCC and paired normal tissue (upper). The middle and bottom dot plots represent AP activity and gene expression normalized by log_2_FC. Promoters ranked by log_2_FC normalized promoter activity. **(C)** Venn diagram showing 64 genes with APs concurrently harboring both upregulated (Up) and downregulated (Down) promoters. **(D)** UCSC genome browser screenshot showing mean read count of prmtr.27493 and prmtr.27494 at the *RARA* gene locus in HCC (red track) and normal tissues (blue track). **(E)** The boxplot showing the expression of gene *RARA* in tumor and normal was nearly the same. ns: not significant. p-value > 0.05 (ANOVA, p-value = 0.88). **(F)** The boxplot showing promoter activity of prmtr.27493 was significantly higher in HCC samples. ***p-value <0.001 (ANOVA, *p*-value = 1.35e-04). **(G)** t-SNE plot showing normal (blue dots) and HCC (red dots) samples can be clustered by activities of all APs. **(H)** Bubble plots showing the enriched biological processes by gene ontology (GO) analysis of gene groups in **(C)**. Bubble color represents the log_2_(*p*-value) while the bubble size represents enriched gene counts.

Next, we aimed to identify the APs based on the above calculated promoter activities in HCC. To this end, we firstly defined the APs according to the gene expression and promoter activity. As shown in **Figure 2A**, the promoters with differential promoter activities (1.2-fold changes), but whose gene expression was not significantly changed, were defined as APs. A total of 855 APs from 709 genes were filtered by this screening of promoter activity and gene expression (**Figure 2B** and **Supplementary Table 1**). The heatmap with the normalized promoter activities and the plot with promoter activity and gene expression changes were drawn to show the properties of all 855 APs (**Figure 2B**). Sixty-four genes with both upregulated and downregulated APs could be good examples of switch usages of promoters: when one promoter is suppressed, another nearby one is activated (**Figure 2C** and **Supplementary Table 2**). For APs, while the gene expression changes were not obvious, the promoter activity changed significantly. For example, in the proto-oncogene *RARA*, the activity of prmtr.27493 was remarkably higher in the tumor, while the activity of prmtr.27494 remained unchanged in both tumor and normal samples (**Figure 2D**). Compared with normal tissues, the gene expression of *RARA* was unchanged (**Figure 2E**), while the promoter activity of prmtr.27493 was significantly enhanced in HCC (**Figure 2F**). When reviewing the genes with APs, we noticed that there are several other known cancer-associated genes, such as *MET* (42) (**Supplementary Figure 2A**), *MICU1* (43) (**Supplementary Figure 2C**), and *SLC19A1* (44) (**Supplementary Figure 2D**). The abnormally upregulated promoter activities in HCC may lead to the changes of the CDS region and produce new protein subtypes, as reported (12). For example, the upregulated prmtr.14927 in *MET* may lead to the accumulation of a 960-aa protein isoform that lacks the SEMA domain in HCC (**Supplementary Figure 2B**). The t-SNE analysis suggested that APs activity could obviously distinguish tumor tissues from normal tissues (**Figure 2G**). A similar result was obtained by PCA (**Supplementary Figure 2E**). We further investigated the association between AP and corresponding transcript isoforms. As shown in **Supplementary Figure 2F**, 60.5% (517/855) of APs only regulate one transcript isoform, and 39.5% (388/855) of APs regulate two or more isoforms. The expression levels of transcription isoforms were positively correlated significantly with the promoter activities (*R* = 0.65, *p* < 2.2e-16; **Supplementary Figure 2G**). For APs with only one transcript isoform, differences in promoter activities could lead to 42.4% (219/517) of transcript isoform with significant expression changing (*p*-value < 0.05) and 31.5% (163/517) of transcript isoform with expression changing (|Fold Change| > 1.2). The remaining promoters may have little effect on the transcript expression changing (**Supplementary Figure 2H**). For APs with multiple transcription isoforms, an AP was identified as an AP with major significant differentially expressed isoforms if one transcription isoform has the most significant change (*p*-value < 0.05), and an AP was identified as an AP with major differentially expressed isoforms if one transcription isoform has the most expression change (|Fold Change| > 1.2). The results demonstrated that 53% of multiple isoform promoters were classified as AP with major significant differentially expressed isoforms and 33.7% (114/388) were classified as AP with major differentially expressed isoforms (**Supplementary Figure 2I**). Further functional enrichment analysis showed that genes with both upregulated or downregulated APs were enriched in cancer-related ontologies, such as ERBB signaling pathway and positive regulation of cell migration (**Figure 2H**). All the results suggested that the usage of APs may play a significant role in the cellular transformation and progression of HCC.

### The Activities of AP Were Significantly Correlated With DNA Methylation Status

**Figure 3:**
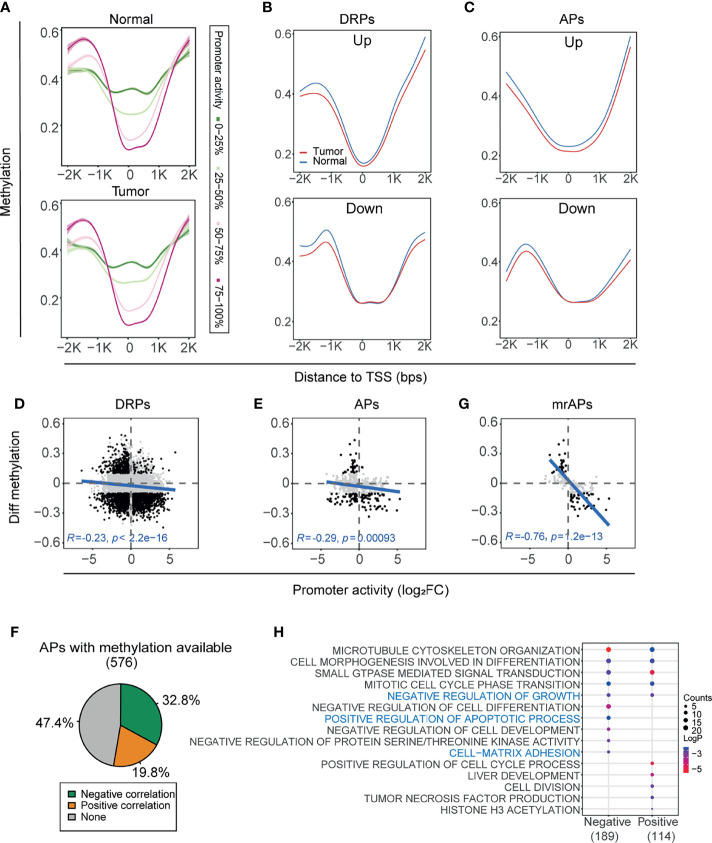
The activity of AP was significantly correlated with DNA methylation status. **(A)** Methylation levels of CpGs within ±2 kbps relative to TSS were assessed in four groups classified by the quartiles of promoter activities. Methylation profile was smoothed by gam (Generalized Additive Models). Green to red represents the promoter activity levels from 0 to 100%. **(B, C)** The methylation profile showing mean methylation levels of TSS nearby region ( ± 2kb) of the DRPs **(B)** and APs **(C)**. Upregulated (top) and downregulated (bottom) promoters were shown separately. The blue line and red line represent normal and HCC samples, respectively. **(D, E)** Scatter plots showing the correlation between differential methylation (HCC – normal) and promoter activity by normalized change fold for DRPs **(D)** and APs **(E).** The representative CpG sites were filtered from the ±1kb upstream and downstream of TSS (see also *Materials and Methods*). Black dots represent the differentially regulated promoters with significant methylation changes (|Diff. methylation| > 0.1); only these black dots were used for the Pearson correlation test. **(F)** The proportions of correlation categories between the promoter activities of APs and their methylation status are shown in the pie chart. Negative, positive, and no correlations are colored by green, orange, and gray, respectively. **(G)** Similar to **(D, E)**, but for methylation regulated APs (mrAPs). **(H)** Bubble plots showing the enriched biological processes by gene ontology (GO) analysis of APs with negative and positive correlations in **(F)**.

DNA methylation, one of the most abnormal epigenetic modifications in cancers, is known to regulate transcriptional activity (45). To explore whether DNA methylation regulates the usage of APs in HCC, we first obtained the methylation status of the same paired tissues based on Infinium Human Methylation 450 K BeadChip (Illumina 450 K array) of GSE77276 (28). Then, all the promoters were classified into four groups by the quartiles of promoter activities, and the overall CpG methylation status of the four groups in the region (−2kb–2kb) of transcription start sites (TSSs) was calculated in either cancer or normal samples (**Figure 3A**). Notably, in the region 1,000 bps around TSS, the higher promoter activity correlated with lower methylation status in both tumors and normal tissues (**Figure 3A**). Next, we compared the methylation status of DRPs. In the vicinity of TSS, promoters upregulated in tumors have lower methylation status than normal tissues, whereas the downregulated promoters would be inclined to have a higher methylation status in tumors (**Figure 3B** and **Supplementary Figures 3A, B**, upper). Last, we focused on the genes with APs, and a similar correlation between the promoter activity and methylation was observed in both upregulated and downregulated APs (**Figure 3C** and **Supplementary Figures 3A, B**, lower).

As shown in **Figures 3A–C**, the most significant changes in CpG methylation were located upstream and downstream of 1,000 bps to TSS, and we next focused on these regions to examine the correlation of methylation of each CpG with promoter activity. As shown in the illustrative cartoon (**Supplementary Figure 3C**), we calculated the correlation between each CpG methylation and the related promoter activity and selected the CpG site with the most significant *p*-value to represent the CpG methylation status of the promoter. The activities of 37.0% (2,468/6,674) of DRPs were significantly negatively correlated (green) with their methylation status, and 23.0% (1,536/6,674) were positively correlated (orange) (**Supplementary Figure 3D**). A negative correlation between the changes of gene expression and methylation in DEGs could be observed (**Supplementary Figures 3E, F**). The correlation test results showed that negative correlation between promoter activity and methylation status in DRPs was stronger than gene expression (*R* = −0.23, *p*-value < 2.2e-16; **Figure 3D** and **Supplementary Figure 3G**), as was the correlation results in APs (*R* = −0.29, *p*-value = 0.00093; **Figure 3E** and **Supplementary Figure 3H**). When examining the correlation of promoter activity and methylation for each AP, we observed that the activities of more than half of APs were significantly correlated with their methylation status, of which 32.8% (189/576) were negatively correlated (green) and 19.8% (114/576) were positively correlated (orange) (**Figure 3F** and **Supplementary Table 3**). Previous studies showed that gene expression could be silenced by hypermethylation of promoters (16–18) and the overexpression of oncogenes (19) could be associated with hypomethylated regions. We then termed the 189 APs with negative correlations as methylation-regulated APs (mrAPs). Consistent with our expectation, the negative correlation in mrAPs was significant (*R* = −0.76, *p*-value = 12e-13; **Figure 3G** and **Supplementary Figure 3I**). Next, we further investigated the association between mrAPs and the corresponding transcript isoforms (**Supplementary Table 4**). When comparing the transcription isoform status of mrAPs to the ones of APs, we observed that both the frequency of significantly differentially expressed isoform for promoters with one transcript isoform (56.3% of mrAPs versus 42.4% of APs) and the frequency of major significantly differentially expressed isoforms for promoters with multiple transcript isoforms (mrAPs 57.8% vs. APs 53%) in mrAPs were higher than in APs (**Supplementary Figures 3J–L**). Gene ontology analysis revealed that those methylation-associated promoters were enriched for ontologies known to be deregulated in HCC, such as negative regulation of growth, positive regulation of apoptotic process, and cell matrix adhesion (**Figure 3H**). Those results demonstrated that usage of APs may be regulated by DNA methylation in HCC.

### The Methylation Regulated APs Could Be Used as Tumor Diagnostic Markers

**Figure 4:**
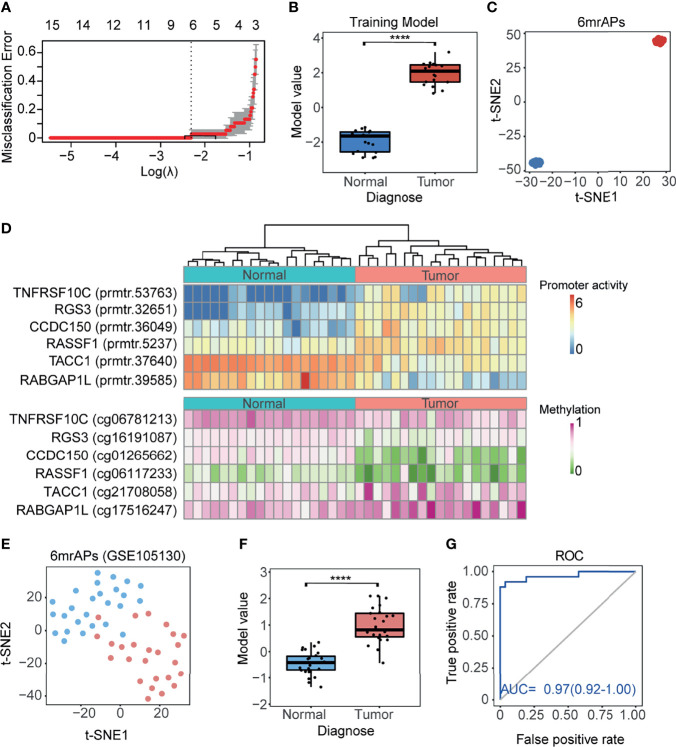
A good performing HCC diagnosis model was generated using mrAPs. **(A)** The cross-validation fit curve was calculated by the LASSO regression using promoter activities of all mrAPs, and six mrAPs were filtered to generate a diagnostic model. **(B)** The boxplot showing the significant different model scores of 19 paired tumors and normal samples based on the diagnostic model of 6mrAPs. **** *p*-value <0.0001 (Wilcoxon test, *p*-value = 5.7e-11) **(C)** t-SNE plot showing normal (blue dots) and HCC (red dots) samples can be significantly distinguished by the promoter activities of six mrAPs. **(D)** The upper heatmap showing the promoter activities of six mrAPs, the lower heatmap showing the screened CpG methylation status of six mrAPs. Samples are arranged in consistent order in both diagrams. **(E)** t-SNE plot showing normal (blue dots) and HCC (red dots) samples could also be grouped by the activities of six mrAPs in the independent test dataset of GSE105130. **(F)** Boxplot showing the significantly different model scores of HCC and normal sample of the test dataset of GSE105130. ****p-value <0.0001 (Wilcoxon test, *p*-value = 2.8e-11) **(G)** ROC curve showing the performance and prediction accuracy of the diagnostic model in the test dataset of GSE105130.

As shown above, the correlation of promoter activity and DNA methylation in APs was observed, and we then explored whether the activities of mrAPs could serve as diagnostic markers. We evaluated it by the least absolute shrinkage and selection operator (LASSO) models. By the LASSO regression model, six out of 189 mrAPs were selected to generate a diagnostic model (**Figure 4A**). The diagnostic model scores of tumors and normal tissues were significantly different (**Figure 4B**), and the dimensional-reduction analysis based on the promoter activities of the six mrAPs showed that the classifier was particularly effective (**Figure 4C**). The six mrAPs were clustered into four upregulated mrAPs (prmtr.53735 of *TNFRSF10*, prmtr.32651 of *RGS3*, prmtr.36049 of *CCDC150*, and prmtr.5237 of *RASSF1*) and two downregulated mrAPs (prmtr.37640 of *TACC1* and prmtr.39585 of *RABGAP1L*) by promoter activities (**Figure 4D**, upper; **Table 1** and **Supplementary Table 5**). The CpG methylation status of the six mrAPs showed an opposite trend when compared to the promoter activities (**Figure 4D**, lower; **Table 1** and **Supplementary Table 5**).

**Table 1:** Promoter activity and methylation alterations of mrAPs in the HCC diagnosis model.

AP	Gene	Probe ID	Probe Dist.to TSS	Diff. promoter activity	Diff. methylation
prmtr.53763	*TNFRSF10C*	cg06781213	−498	3.04	−0.11
prmtr.32651	*RGS3*	cg16191087	−245	2.67	−0.09
prmtr.36049	*CCDC150*	cg01265662	−232	1.90	−0.28
prmtr.5237	*RASSF1*	cg06117233	−718	1.18	−0.13
prmtr.37640	*TACC1*	cg21708058	−102	−2.18	0.11
prmtr.39585	*RABGAP1L*	cg17516247	−331	−2.97	0.09

AP, alternative promoter; mrAPs, methylation-regulated APs; Dist., distance; Diff., Difference (Tumor-Normal).

Two other independent public datasets of GSE105130 (30) and GSE55758 (29) were then further used as test datasets to assess the diagnostic model. The promoter activities of six mrAPs were successful in discriminating tumor from normal using t-SNE (**Figure 4E** and **Supplementary Figure 4A**). The classify model yielded significant differences between tumor and normal samples (**Figure 4F** and **Supplementary Figure 4B**). The AUC score of 0.97 (GSE105130) and 0.95 (GSE55758) indicated the good performance of our classifier in both test datasets (**Figure 4G** and **Supplementary Figure 4C**). In this section, we constructed a tumor diagnostic model based on promoter activities of six mrAPs with significant diagnostic effects, which indicated that the promoter activity of mrAPs could be valuable in tumor diagnosis.

### The Methylation Status of APs Could Be Used as a Prognostic Indicator in HCC

**Figure 5:**
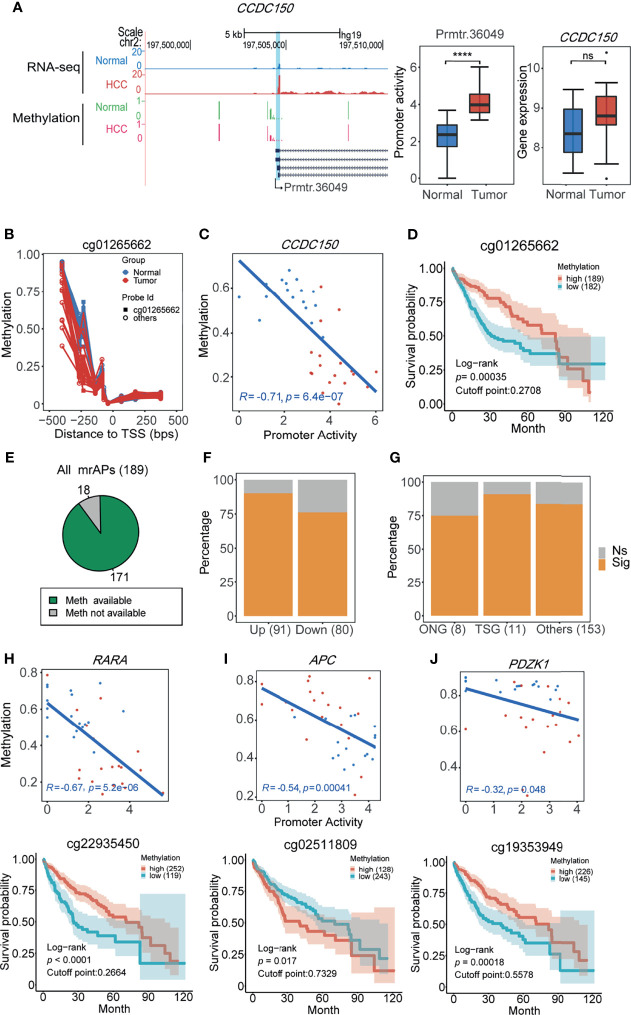
The methylation status of APs predicts patient survival in HCC. **(A)** UCSC genome browser screenshot showing mean read count (top 2 tracks) and 450K methylation beta values (bottom 2 tracks) at the *CCDC150* gene locus of HCC (red) and normal tissues (blue or green). The statistical results are shown in the middle (boxplot for promoter activity, ****p-value <0.0001 (ANOVA, *p*-value = 1.14e-07)) and right (boxplot for gene expression, ns: not significant. p-value > 0.05 (ANOVA, p-value = 0.12)). **(B)** Methylation beta values of ±500 bps relative to TSS (prmtr.36049). Normal and tumor samples are colored by green and red dots, dots from the same sample were connected by lines. Cg01265662 with the lowest *p*-value for the correlation test was marked and screened for calculation in **(C)** and **(D)**. **(C)** The scatter plot showing the negative correlation between promoter activities of prmtr.36049 and methylation beta values of cg1265662 in HCC (red) and normal (blue) samples (Pearson correlation). **(D)** The 10-year overall survival curve of methylation levels of cg1265662 in TCGA-LIHC patients in the high and low methylation cohort, showing that methylation of cg1265662 was significantly associated with survival in HCC. **(E)** The pie plot showing the majority of mrAPs with methylation values available in TCGA-LIHC. **(F)** The proportion of mrAPs with significant prognostic CpG methylation sites in upregulated and downregulated ones. **(G)** The proportion of genes with significant prognostic CpG methylation sites in mrAPs grouped by oncogenes (ONG), tumor suppressor genes (TSG), and others. **(H–J)** The correlations between promoter activity and methylation levels (upper) similar to **(C)** and corresponding survival curve of CpG sites (bottom) similar to **(D)** of *RARA* **(H)**, *APC* **(I)**, and *PDZK1* **(J)** were shown, respectively.

It has been reported that promoter activity could be used as prognostic markers in gastric cancer and renal cancer (11). In our study, approximately half of the promoter activity changed, which was likely due to the alteration of methylation status. We next asked whether the methylation status of APs predicts patient survival in HCC. In order to do this, The Cancer Genome Atlas (TCGA) 450K data of HCC patients with the prognostic information were used for survival analysis. We first focused on the methylation status of the above six mrAPs in the LASSO diagnostic model. As shown in **Figure 5A**, compared with normal samples, the gene expression of *CCDC150* in tumor samples was not significantly changed, but the activities of prmtr.36049 were notably increased. By comparing the methylation levels of the paired samples, a lower methylation level in *CCDC150* in tumor samples was observed (**Figure 5B**). Further analysis revealed that the methylation values and promoter activity of *CCDC150* were well negatively correlated (*R* = −0.71, *p* = 6.4e-7), and the probe with the minimal *p*-value is cg01265662 (**Figure 5C**). The methylation values of cg01265662 were then divided into two clusters based on optimal cutoffs and the length of patient survivals was compared (*p*-value = 0.00035, **Figure 5D**). The higher the methylation level of cg01265662, the better prognostic result was observed. These results indicated that the CpG methylation status of prmtr.36049 of *CCDC150* may be used as a prognostic factor in HCC. We further investigated the other five mrAPs and observed that the CpG methylation status of *RASSF1*, *TACC1*, and *RABGAP1L* could also possess prognostic values (**Supplementary Figure 5A**).

We next analyzed how many mrAPs have prognostic methylation markers in HCC. Among 189 mrAPs, 171 with available probe methylation values from TCGA were used for further analysis (**Figure 5E**). The results showed that 83.63% (143/171) of mrAPs had prognostic methylation markers, with 90.11% (82/91) of upregulated mrAPs and 76.25% (61/80) of downregulated mrAPs (**Figure 5F** and **Supplementary Table 6**). It contained ten switch-usage AP genes, with the example of *ARAP1* exhibited in **Supplementary Figures 5B–E**. The ONGene and TSGene already had catalogs genes closely associated with tumorigenesis and development. Six of eight oncogenes (ONG) and 10 of 11 tumor suppressor genes (TSG) had methylation markers (**Figure 5G**). The higher expression of the oncogene *RARA* might be regulated by the hypomethylation, and the lower methylation status predicted a worse clinical outcome (**Figure 5H**), while for TSG *APC*, the lower methylation status predicts a better prognostic result (**Figure 5I**). Among the genes from the ONG and TSG lists, some may also play a role in cancers. For example, *PDZK1* (**Figure 5J**), which is related to cancer progression, had been reported in different kinds of cancers, such as gastric cancer (46), renal cell carcinoma (47), and breast cancer (48). However, *PDZK1* played a different or even opposite role in other tumors. In our study, the lower methylation accompanied by higher expression status had a worse survival trend, which implied that *PDZK1* may harbor oncogenic activity in HCC. Taken together, these results demonstrated that CpG methylation status of APs may be used as a prognostic marker by altering the activities of the promoters and provided a new perspective for understanding the underlying mechanisms of cancer development.

### Validation of Promoter Activity and Methylation Status by RNA-seq and WGBS

**Figure 6:**
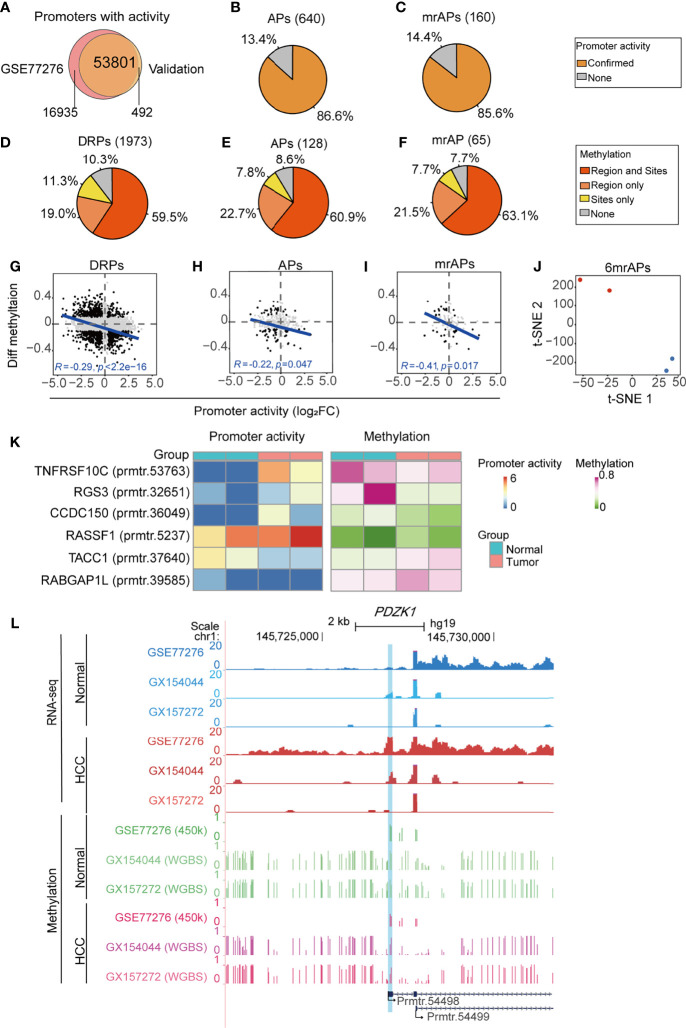
Validation of promoter activity and methylation status by RNA-seq and WGBS. **(A)** Venn diagram showing the overlap of promoters with activities in our validation dataset of RNA-seq and public dataset of GSE77276. **(B, C)** The pie chart showing the percentage of all identified APs **(B)** and mrAPs **(C)** in GSE77276 with differential promoter activities being confirmed by our validation dataset of RNA-seq. **(D–F)** The pie chart showing the percentage of all identified DRPs **(D)**, APs **(E),** and mrAPs **(F)** in GSE77276 with differential methylation status being confirmed by our validation dataset of WGBS. Changes of methylation of CpG site (site) with over 0.1 in both public 450K data and our validation WGBS data, and also with the same alteration trends, would be regarded as confirmed. The promoter region (region) mean methylation was adopted for testing if the methylation of a CpG site is not available in the WGBS dataset. **(G–I)** Scatter plots showing the correlation between differential methylation (HCC – normal) and promoter activity by normalized change fold for DRPs **(G)**, APs **(H)**, and mrAPs **(I),** similar to **Figures 3D, E, G**, but in our validation datasets of RNA-seq and WGBS. **(J)** The t-SNE plot showing the normal (blue dots) and HCC (red dots) samples in our validation dataset could be grouped by the promoter activities of six mrAPs used in the diagnostic model in **Figure 4E**. **(K)** Heatmap showing the promoter activities and methylation status of six mrAPs in our validation data. **(L)** UCSC genome browser screenshot showing the promoter activities and methylation of gene *PDZK1* in both public dataset of GSE77276 and our validation datasets. GSE77276 RNA-seq tracks represent mean read counts of 19 samples, and validation dataset RNA-seq tracks represent read counts for HCC or normal samples from GX154044 and GX157272 separately. GSE77276 450K tracks represent the mean methylation value of 19 samples, and validation dataset WGBS tracks represent methylation ratio for samples from GX154044 and GX157272 separately.

To systematically verify the above results, we collected four samples (two HCC and two paired para-cancer tissues) for RNA-seq and WGBS. Mapping statistical information of RNA-seq and WGBS data are shown in **Supplementary Table 7**. A total of 54,293 promoters with activities (**Figure 6A**) were obtained, suggesting that the usage of promoters in cancer may have a sample or subtype heterogeneity (9). We first verified the APs and mrAPs activity changes in our validation data by the criteria of 1.2-fold change between tumor and normal tissues. The results showed that activity changes of 86.6% (554/640) APs and 85.6% (137/160) mrAPs identified by a public dataset could be confirmed in at least one pair of our validation data (**Figures 6B, C** and **Supplementary Figures 6A, B**). We next aimed to verify the methylation status of promoters using WGBS. We calculated the genome-wide CpG methylation and the average methylation levels of the TSS region of promoters. The methylation level of the TSS region gradually decreased with the increase of the promoter activity level in both pairs of HCC patients (**Supplementary Figure 6C**). Then, we focused on verification of the CpG sites selected by the correlation test in the public data previously. A differential methylation ratio over 0.1 with the same alteration trends in both public 450K data and our validation WGBS data would be regarded as confirmed. By WGBS, the average methylation status of the region was more effective for the representativeness of the promoter methylation status and could compensate for the lack of sites and deviations caused by a single methylation site. Then, we further calculated the mean methylation of the promoter regions (−1kb–1kb) and compare it to the methylation status of the selected CpG sites above. So, we next used mean methylation (sequencing reads covered all the samples) of promoter region for the sites without enough methylation information in WGBS for further analysis. About 89.7% (1,769/1973) of the significant methylation changes of the available DRPs CpG sites were verified in our samples (**Figure 6D**). Among them, the confirmed ratios for APs and mrAPs were 91.4% (117/128) and 92.3% (60/65), respectively (**Figures 6E, F**). The higher confirmed ratios (90.6% for DRPs, 96.6% for APs, and 95.7% for mrAPs) were achieved when the criteria of significant methylation changes were enhanced to 0.2 (**Supplementary Figures 6D–F**). The negative correlation between the changes of promoter activity and the regional methylation level in DRPs (*R* = −0.29, *p*-value = 2.2e-16; **Figure 6G**) and APs (*R* = −0.22, *p*-value = 0.047; **Figure 6H**) were also confirmed in our validation. Consistent with our expectation, the correlation coefficient in mrAPs was higher (*R* = −0.41, *p*-value = 0.017; **Figure 6I**). A similar negative correlation was also obtained using selected CpGs sites (**Supplementary Figures 6G–I**). As shown above, our RNA-seq and WGBS data powerfully verified the negative correlation of promoter activity and methylation status in HCC.

In addition, tumor samples could be successfully distinguished from normal samples by the activities of six mrAPs using t-SNE, which confirmed the accuracy of the above diagnostic model (**Figure 6J**). Heatmap showed the activity trends of the six mrAPs used in the diagnostic model, which were consistent with the above results (**Figure 6K**). The methylation status of six mrAPs in our validation data showed a similar changing pattern with **Figure 4D** (**Figure 6K**). Finally, examples mentioned above such as *PDZK1* were examined in the UCSC genome browser (**Figure 6L** and **Supplementary Figures 6J**). As shown in **Figure 6L**, the promoter (prmtr.54498) of the tumor samples was higher than normal, both in the public data and our two samples. The CpG methylation status of prmtr.54498 was lower in the tumor samples, and our WGBS data showed a more pronounced effect. The correlation between the promoter activity and methylation status, including our validation data, is shown in **Supplementary Figure 6J**. Another example of *CCDC150* is shown in **Supplementary Figures 6K, L**. In addition, these observations were further verified by an independent validation based on public WGBS and RNA-seq datasets (31) of the paired tumor and normal samples from two patients (**Supplementary Figures 6M–R**). Through the comprehensive analysis of our validation data and independent public datasets of RNA-seq and WGBS, we further confirmed the effects of aberrant DNA methylation on the usage of APs in HCC from a genome-wide perspective, which provides a new insight into the exploration of tumor mechanisms.

## Discussion

Promoters are one of the key factors that regulate gene expression. Recent studies showed that the differential activities of promoters had a significant impact on the cancer transcriptome and contribute to the cellular transformation of cancer (11, 12). Genome-wide promoter analysis methods such as H3K4me3 ChIP-seq, CAGE, and RNA long-read sequence had a limitation in the numbers of publicly available datasets. In this study, we applied “proActiv”, an R package quantification promoter activity based on the widely used RNA short-read sequencing. Promoter activity inferred by “proActiv” has been advocated to have high consistency with other technologies (11, 12). So far, this study provides the first systematic study of genome-wide promoters usage in HCC. Compared to the gene expression, promoter activity successfully distinguished the tumor samples from normal by either t-SNE or PCA, which suggested an advantage by promoter activity in identifying the differences between tumor and normal samples. In addition, some cancer-related ontologies enriched only in genes with differentially regulated promoters (DRPGs) implied that promoter analysis may provide more information to the potential mechanism of tumorigenesis and development.

DEGs of HCC have been emphasized in previous studies, but only a few studies focused on the non-differential genes (non-DEGs). In this study, we focused on the promoters that belong to the non-DEGs. DRPs were considered as an AP if they were derived from non-DEGs. We identified 855 APs from 709 genes, among which are several known cancer-associated genes, such as *RARA*, *ARAP1*, and *MET*. *MET*, a prototypical receptor tyrosine kinase, has been reported in several cancers and regulates many physiological processes including proliferation, morphogenesis, and survival (42). In our study, the promoter activity of the N short-truncated isoform was significantly increased in HCC patients, which may lead to abnormal SEMA domain lacking protein accumulation. The abnormally increased expression of the N short-truncated *MET* isoform had also been observed in gastric cancer (12). Further studies are required to determine how the abnormal SEMA-lacking protein accumulation plays a role in tumor development.

Few studies have aimed to determine the potential mechanism of regulation in the usage of APs. DNA methylation is one of the most deeply studied epigenetic regulatory potential mechanisms. The canonical mechanisms of transcript silencing caused by hypermethylation include the following: (1) hypermethylation interferes with transcription factor binding, (2) methylated DNA-binding protein (MDBP) prevents the binding of transcription factors to target sequences in the promoter, and (3) hypermethylation changing chromatin structure leads to tighter chromatin structure and transcriptional inactivation (15, 49). This is the first systematic study focusing on the relationship between methylation status and promoter activities in APs. In our study, there are approximately 53% APs activity in cancers likely to be regulated by DNA methylation, among which 62% show canonical negative correlations. A positive correlation had also been reported in a selection of contexts (50). However, its potential mechanism needs further exploration. Taken together, our results indicated that the aberrant methylation states play a critical role in the precision usage of APs in HCC.

We next focused on the diagnostic and prognostic values of methylation-regulated APs (mrAPs). Based on the LASSO regression model, six out of 189 mrAPs were selected to generate a diagnostic model, which works well in both the training and testing datasets. For the six mrAPs in the diagnostic model, five mrAPs (prmtr.53763 of *TNFRSF10C*, prmtr.32651 of *RGS3*, prmtr.36049 of *CCDC150*, prmtr.37640 of *TACC1*, and prmtr.39585 of *RABGAP1L*) belong to a multiple isoform mrAP with major significantly differentially expressed isoforms. prmtr.5237 of RASSF1 belongs to promoters regulating one transcript isoform with a significant expression change. All of these observations may highlight the more significant effect of multiple-isoform APs with major significantly differentially expressed isoforms on the development of HCC. TNFRSF10C works as an antagonistic receptor that protects cells from TRAIL-induced apoptosis. The copy number variation of *TNFRSF10C* and the downregulation of protein TNFRSF10C have been reported to be associated with colorectal cancer metastasis (51, 52). In our work, the activity of prmtr.53763 in *TNFRSF10C* was significantly upregulated and the role of transcript isoform is regulated by this promoter in tumors and needs to be explored in future studies. RGS3 is a GTPase-activating protein that inhibits G-protein-mediated signal transduction and associated with tumor cell proliferation and migration in glioma (53) and gastric cancer (54). Our study observed that *GRS3* promoter activity (prmr.32651) is significantly and steadily upregulated in HCC. RASSF1 plays an important role in the occurrence and process of malignant tumors. It contains two well-studied subtypes, RASSF1A and RASSF1C, due to AP usage. Our research showed that hypermethylation of the *RASSF1A* promoter (prmr.5239) associated with the downregulation of promoter activity and tended to have poorer cancer survival, which was consistent with previous studies (55–57). In addition, the hypomethylation of *RASSF1C* promoter (prmtr.5237) was associated with the upregulation of promoter activity, which could serve as an oncogene in both our study and previous research (58). By interacting with a variety of complexes, *TACC1* participates in tumorigenesis and development. Abnormal *TACC1* regulation plays an important role in the occurrence and development of multiple myeloma including breast cancer (59), gastric cancer (60), and ovarian cancer (61). Our research demonstrated that the methylation status of the *TACC1* promoter region is significantly related to promoter activity, which implies the new roles of *TACC1* in liver cancer. *RABGAP1L* is a protein coding gene that is functionally involved in endocytosis and intracellular protein transport by regulating the activity of GTPases (62). *CCDC150* is a protein coding gene with multiple transcripts. We reported that the CpG methylation status of *CCDC150* and *RABGAP1L* could have prognostic values in HCC, which linked the functions of these two genes to cancer development.

It has also been reported that promoter activity could be used as a prognostic marker in gastric cancer and renal cancer (11). DNA methylation could potentially function as a tumor biomarker with high stability and high specificity. Traditionally, DNA methylation studies were mainly based on the DEGs, and the gene promoter regions are usually located upstream and downstream of the most distal transcription start site. However, in reality, more than half of the genes have one or more transcription start sites, and a large amount of gene-related methylated regions are being overlooked. In our study, 83.63% (143/171) of mrAPs had at least one associated methylation site that could be used to predict clinical outcomes. Methylation of four promoters in the diagnostic model and several known oncogenes or tumor suppressor genes’ promoters were included.

*RARA* has been reported to promote tumor progression in breast cancer (63), acute promyeloid leukemia (64), and liver cancer (65). In our study, the methylation level of the promoter (prmtr.27493) of *RARA* in HCC was significantly decreased, with the increase of promoter activity (65, 66). It is likely that the full-length transcript was overexpressed in HCC, which may promote the development of the tumor. The tumor suppressor gene (TSGene) *APC* has been most studied in colorectal cancer (67), and its role in liver cancer has also been reported (68–70). In contrast to *RARA*, hypermethylation of a promoter (prmtr.29535) inhibits the transcription and may contribute to the intensification of tumor progression. The oncogenic activity of *RARA* and tumor suppressor activity of *APC* observed in our study supported their roles that were reported in previous research. In addition, there are some cancer-associated genes from the oncogene and TSGene lists. *PDZK1* plays a different or even opposite role in different tumors. *PDZK1* acts as a tumor suppressor in gastric cancer and renal cancer, but in esophageal adenocarcinoma, breast cancer, and multiple myelomas (MMs), the overexpression of *PDZK1* promotes cancer development or drug resistance (71–73). We found that *PDZK1* promoter activity was significantly increased in the HCC, and was significantly correlated with methylation status, which showed that the lower methylation group of patients would have a worse prognosis. Our results suggested that *PDZK1* may harbor oncogenic activity in HCC.

Finally, we used RNA-seq and WGBS in HCC patients to perform a comprehensive verification of our study. The significant changing of promoter activities of 86.6% (554/640) APs and 85.6% (137/160) mrAPs could be confirmed in our validation dataset. A majority of the selected 450K CpGs with significantly changed methylation sites could be confirmed in our WGBS validation dataset, especially in mrAPs. A negative correlation between the change of promoter activity and the methylation variation implied that methylation may regulate the usage of APs in HCC. In addition, both promoter activity and the methylation status of the six methylation-regulated APs used in the diagnostic model could also be verified in the validation data. We extended our validations to two other independent pairs of liver cancer and matched normal samples from the public dataset (31). Some limitations exist in our study due to the small sample size, and more WGBS samples would be investigated in our future studies. However, the relationship between promoter activities and methylation changing of APs in cancer and normal samples could be validated on a genome-wide scale by paired WGBS and RNA-seq data. All in all, our results suggested that the study of APs and their methylation status can have a general application in liver cancer.

## Conclusion

Our study demonstrated that promoter activity was more effective for HCC recognition than gene expression, and the usage of APs has a significant influence on the cancer transcriptome. Furthermore, the precise usage of APs could be regulated by DNA methylation in HCC, which would have a great effect on the comprehensive understanding of the tumorigenesis mechanism. Finally, based on methylation-regulated APs, our study provided an effective potential approach for cancer screening and treatment. Taken together, our study provided a new perspective on transcription regulation and contributed to the cellular transformation of cancer.

## Data Availability Statement

The RNA-seq and WGBS raw sequence data generated in this paper have been deposited in the Genome Sequence Archive (74) in National Genomics Data Center (75), China National Center forBioinformation / Beijing Institute of Genomics, Chinese Academy of Sciences, under accession number HRA001330 that are publicly accessible at https://ngdc.cncb.ac.cn/gsa-human/browse/HRA001330. The processed data related to WGBS, RNA promoters’ activity and gene expression are publicly deposited at jianguoyun.com. (https://www.jianguoyun.com/p/Da8nQ6UQ7cDxCRiKhqUE). The source data for all the figures and supplementary figures are available in **Supplementary Table 8**. The public datasets presented in this study can be found in online repositories. The names of the repository/repositories and accession number(s) can be found in the article/**Supplementary Material**.

## Ethics Statement

The studies involving humans were approved by the Ethics Committee of Guangxi Medical University Cancer Hospital. The studies were conducted in accordance with the local legislation and institutional requirements. The participants provided their written informed consent to participate in this study.

## Author Contributions

XH and YD conceived and designed the study. QW and BX collected the tissue sample and performed sequencing. YD, XL, SW, BJ, and RL performed the data analysis. YD and XL drafted the manuscript. XH revised the manuscript. XH and QW supervised the study. All authors contributed to the manuscript and approved the submitted version.

## Funding

The project was supported by Fundamental Research Funds for the Central Universities, HUST (No. 2021GCRC073). Guangxi Key Research and Development Program (AB18126055 and AB20297009).

## Conflict of Interest

The authors declare that the research was conducted in the absence of any commercial or financial relationships that could be construed as a potential conflict of interest.

## Publisher’s Note

All claims expressed in this article are solely those of the authors and do not necessarily represent those of their affiliated organizations, or those of the publisher, the editors and the reviewers. Any product that may be evaluated in this article, or claim that may be made by its manufacturer, is not guaranteed or endorsed by the publisher.

## Supplementary Material

Supplementary Figure 1 Comparative analysis of promoter activity and gene expression in HCC. Related to **Figure 1**. **(A)** The number of promoters with activities in HCC per gene (left); number of differentially regulated promoters (DRPs) with activities in HCC per gene. **(B–C)** Volcano plot showing the log_2_ (fold change) in gene expression **(B)** and promoter activity **(C)** in HCC and adjust normal tissues. **(D)** Principal component analysis (PCA) clustering the sequenced samples by FPKM for all genes or DEGs and by promoter activities for all promoters or DRPs. Samples were colored by sample types (dark red: HCC; dark blue: adjacent normal tissue).

Supplementary Figure 2 Examples of alternative promoters (APs). Related to **Figure 2**. **(A)** UCSC genome browser screenshot showing mean read count of prmtr.14925 and prmtr.14927 at the *MET* gene locus in HCC (red track) and normal tissues (blue track). The boxplot showing the expression of gene *RARA* in tumor and normal was nearly the same. The boxplot showing promoter activity of prmtr.14927 was significantly higher in HCC samples. *****p*-value <0.0001 (ANOVA, *p*-value = 4.57e-05). **(B)** The protein domains of isoforms initiated from prmtr.14925 and prmtr.14927. **(C)** UCSC genome browser screenshot showing mean read count of prmtr.15778 at the *MICU1* gene locus in HCC (red track) and normal tissues (blue track). The boxplot showing the expression of gene *MICU1* in tumor and normal was nearly the same. The boxplot showing promoter activity of prmtr.15778 was significantly lower in HCC samples. *****p*-value <0.0001 (ANOVA, *p*-value = 1.34e-08). **(D)** UCSC genome browser screenshot showing mean read count of prmtr.53841, prmtr.53843, and prmtr.53844 at the *SLC19A1* gene locus in HCC (red track) and normal tissues (blue track). The boxplot showing the expression of gene *SLC19A1* in tumor and normal was nearly the same. The boxplot showing promoter activity of prmtr.53841 was significantly lower in HCC tissues, but prmtr.53843, and prmtr.53844 were significantly higher in HCC samples. *****p*-value <0.0001 (ANOVA, prmtr.53841: *p*-value = 1.16e-04; prmtr.53843: p-value = 7.58e-03; prmtr.53844: p-value = 7.84e-06). **(E)** PCA plot showing normal (blue dots) and HCC (red dots) samples can be clustered by activities of all APs. **(F)** The number of transcripts with same TSSs in HCC per APs. **(G)** Scatter plots showing the correlation between transcript expression (log2FC) and promoter activity (log2FC) of APs. **(H)** The pie chart showing the percentage of significant differential expressed isoforms (cancer versus normal, p < 0.05), differential expressed isoforms (cancer versus normal, |fold-change| > 1.2) and others in APs with only one transcript isoform. **(I)** The pie chart showing the percentage of AP with major differential expressed isoforms (contain one or more transcript isoform express significantly different, p-value < 0.05), APs with major diff isoform (contain one or more transcript isoform express different, |fold-change| > 1.2) and multi transcript isoform influenced APs.

Supplementary Figure 3 Correlation analysis of promoter activity and methylation status. Related to **Figure 3**. **(A)** The methylation profile showing mean methylation levels of TSS nearby region (±2k) of the DRPs **(Top)** and APs **(Bottom)**. Up-regulated and down-regulated promoters were shown separately. The blue line and red line represent normal and HCC samples respectively. Methylation profile was smoothed by 50 bps sliding windows with 25 bps steps. **(B)** The differential methylation (Tumor - Normal) of each window was calculated and plotted corresponding to **(A) (C)** The Schematic illustrates the method to screen the CpG sites with the minimal p-value correlation. Correlation tests between each CpG methylation status and the promoter activity were performed, and the CpG site with the minimal *p*-value was selected. **(D)** The proportions of correlation categories between the promoter activities of DRPs and their methylation status are shown in the pie chart. Negative correlation, positive correlation, and none correlation are colored by green, orange, and grey respectively. **(E–F)** Scatter plots showing the correlation between methylation (HCC – normal) and promoter activity by normalized change fold for DEGs. (G-I) Scatter plots showing the correlation between differential methylation (HCC – normal) and promoter activity by normalized change fold for DRPs **(G)**, APs **(H)** and mrAPs **(I)**. The representative CpG sites were filtered from the ±1k upstream and downstream of TSS (see also Methods). **(J)** The number of transcripts with same TSSs in HCC per mrAP. **(K)** The pie chart showing the percentage of significant differential expressed isoforms (cancer versus normal, p < 0.05), differential expressed isoforms (cancer versus normal, |fold-change| > 1.2) and others in mrAPs with only one transcript isoform. **(L)** The pie chart showing the percentage of mrAP with major differential expressed isoforms, mrAP with major diff isoform (contain one or more transcript isoform express different, |fold-change| > 1.2) and multi transcript isoform influenced mrAP.

Supplementary Figure 4 Effectiveness of the diagnostic model based on 6 mrAPs in another independent test dataset. Related to **Figure 4**. **(A)** t-SNE plot showing normal (blue dots) and HCC (red dots) samples could also be grouped by the activities of six mrAPs in the independent test dataset of GSE55758. **(F)** Boxplot showing the significant different model scores of HCC and normal sample of the test dataset of GSE55758. ***p*-value <0.01 (Wilcoxon test, *p*-value = 0.0011) **(G)** ROC curve showing the performance and prediction accuracy of the diagnostic model in the test dataset of GSE55758.

Supplementary Figure 5 The methylation status of the case with switch promoter activity usage predicts survival of HCC patients. Related to **Figure 5**. **(A)** The 10 years overall survival curve of methylation levels of cg06781213 (related to TNFRSF10C), cg16191087 (related to RGS3), cg06117233 (related to RASSF1), cg21708058 (related to TACC1), cg17516247 (related to RABGAP1L) in TCGA-LIHC patients in high and low methylation cohort. **(B)** UCSC genome browser screenshot showing mean read count (top 2 tracks) and 450 K methylation beta values (bottom 2 tracks) at the *ARAP1* gene locus of HCC (red) and normal tissues (blue or green). The boxplot shows the gene expression in tumor and normal was roughly equal. The activity of prmtr.61499 is significantly lower in HCC tissues, but prmtr.61501 is significantly higher in HCC samples ***p*-value <0.01 (ANOVA, prmtr.61499: *p*-value = 1.48e-03; prmtr.61501: *p*-value = 3.24e-04). **(C)** Methylation beta value of ± 500 bps relative to TSS of prmtr.61499 (upper) and prmtr.61501 (bottom). Normal and tumor samples are colored by green and red dots, dots from the same sample were connected by lines. Cg16695389 and cg07031551 with the lowest *p*-value for the correlation test were marked and screened for calculation in **(D)** and **(E)**. **(D)** The scatter plot showing the negative correlation between promoter activities of prmtr.61499 and methylation beta values of cg16695389 in HCC (red) and normal (blue) samples. A similar negative correlation exhibits between prmtr.61501 and methylation beta values of cg07031551. **(E)** The 10 years overall survival curve of methylation levels of cg16695389 and cg07031551 in TCGA-LIHC patients in high and low methylation cohort, showing both methylations of cg16695389 and cg07031551 was significantly associated with survival in HCC.

Supplementary Figure 6 Comprehensive verification using RNA-seq and WGBS in HCC patients. Related to **Figure 6**. **(A–B)** The pie chart shows in detail the percentage of all identified APs **(A)** and mrAPs **(B)** in GSE77276 with differential promoter activities being confirmed by our validation dataset of RNA-seq. **(C)** Methylation levels of CpGs within ± 2kbps upstream and downstream relative to TSS were assessed in four groups classified by the quartiles of promoter activities. Green to red represents the promoter activities levels from 0 to 100%. Methylation profile was smoothed by gam (Generalized Additive Models). **(D-F)** The pie chart showing the percentage of all identified DRPs **(D)**, APs **(E)** and mrAPs **(F)** in GSE77276 with differential methylation status being confirmed by our validation dataset of WGBS. Changes of methylation of CpG site (site) with over 0.2 in both public 450K data and our validation WGBS data, and also with the same alteration trends, would be regarded as confirmed. The promoter region (region) mean methylation were adopted for testing if the methylation of a CpG site is not available in WGBS dataset. **(G-I)** Scatter plots showing the correlation between differential methylation (HCC – normal) and promoter activity by normalized change fold for DRPs **(G)**, APs **(H)** and mrAPs **(I)** The available above selected CpG methylation value was used to calculate delta methylation. Only these black dots were used for the Pearson correlation test. **(J)** The scatter plot shows the correlation test result by GSE77276 and validation RNA-seq dataset between the activity of *PDZK1* (prmtr.54498) and methylation levels of its selected CpG (cg19353949) methylation or region mean methylation value, normal and tumor samples are colored by blue and red, validation samples are marked in the plot. **(K)** UCSC genome browser screenshot showing the promoter activities and methylation of gene *CCDC150* in both public dataset of GSE77276 and our validation datasets. GSE77276 RNA-seq tracks represent mean read counts of 19 samples, validation dataset RNA-seq tracks represent read counts for HCC or normal samples from GX154044 and GX157272 separately. GSE77276 450k tracks represent the mean methylation value of 19 samples, validation dataset WGBS tracks represent methylation ratio for samples from GX154044 and GX157272 separately. **(L)** The scatter plot shows the correlation test result by GSE77276 and validation RNA-seq dataset between the activity of *CCDC150* (prmtr.36049) and methylation levels of its selected CpG (cg1265662) methylation or region mean methylation value. **(M)** Heatmap shows the correlation of three pairs of promoter activity in GSE70091. **(N)** Heatmap shows the better performance correlation of two pairs of promoter activity in GSE70091 after removing N3 and T3 pairs. **(O)** The pie chart showing the percentage of all identified DRPs, APs and mrAPs in GSE70091 with differential promoter activities being confirmed by validation dataset of RNA-seq. **(P)** The pie chart showing the percentage of all identified DRPs, APs and mrAPs in GSE70091 with differential methylation status being confirmed by validation dataset of WGBS. Changes of methylation of CpG site (site) with over 0.1 in both public 450K data and our validation WGBS data, and also with the same alteration trends, would be regarded as confirmed. The promoter region (region) mean methylation were adopted for testing if the methylation of a CpG site is not available in WGBS dataset. **(Q)** Scatter plots showing the correlation between differential methylation (HCC – normal) and promoter activity by normalized change fold for DRPs, APs and mrAPs in GSE70091 datasets of RNA-seq and WGBS. The available above selected CpG methylation value was used to calculate delta methylation. Only these black dots were used for the Pearson correlation test. **(R)** Heatmap showing the promoter activities and methylation status of six mrAPs in GSE70091.

## References

[1] McGlynn KA Petrick JL El-Serag HB. Epidemiology of Hepatocellular Carcinoma. Hepatology (2021) 73(Suppl 1):4–13. doi: 10.1002/hep.31288 PMC7577946 32319693

[2] Hartke J Johnson M Ghabril M. The Diagnosis and Treatment of Hepatocellular Carcinoma. Semin Diagn Pathol (2017) 34(2):153–9. doi: 10.1053/j.semdp.2016.12.011 28108047

[3] Allemani C Matsuda T Di Carlo V Harewood R Matz M Nikšić M. Global Surveillance of Trends in Cancer Survival 2000-14 (CONCORD-3): Analysis of Individual Records for 37 513 025 Patients Diagnosed With One of 18 Cancers From 322 Population-Based Registries in 71 Countries. Lancet (2018) 391(10125):1023–75. doi: 10.1016/S0140-6736(17)33326-3 PMC5879496 29395269

[4] Wang EA Stein JP Bellavia RJ Broadwell SR. Treatment Options for Unresectable HCC With a Focus on SIRT With Yttrium-90 Resin Microspheres. Int J Clin Pract (2017) 71(11):e12972. doi: 10.1111/ijcp.12972 28758319

[5] Yang JD Mannalithara A Piscitello AJ Kisiel JB Gores GJ Roberts LR. Impact of Surveillance for Hepatocellular Carcinoma on Survival in Patients With Compensated Cirrhosis. Hepatology (2018) 68(1):78–88. doi: 10.1002/hep.29594 29023828 PMC5897179

[6] Kimura K Wakamatsu A Suzuki Y Ota T Nishikawa T Yamashita R. Diversification of Transcriptional Modulation: Large-Scale Identification and Characterization of Putative Alternative Promoters of Human Genes. Genome Res (2006) 16(1):55–65. doi: 10.1101/gr.4039406 16344560 PMC1356129

[7] Marcu KB Bossone SA Patel AJ. Myc Function and Regulation. Annu Rev Biochem (1992) 61:809–60. doi: 10.1146/annurev.bi.61.070192.004113 1497324

[8] Amaar YG Minera MG Hatran LK Strong DD Mohan S Reeves ME. Ras Association Domain Family 1C Protein Stimulates Human Lung Cancer Cell Proliferation. Am J Physiol Lung Cell Mol Physiol (2006) 291(6):L1185–L90. doi: 10.1152/ajplung.00072.2006 16891396

[9] Qamra A Xing M Padmanabhan N Kwok JJT Zhang S Xu C. Epigenomic Promoter Alterations Amplify Gene Isoform and Immunogenic Diversity in Gastric Adenocarcinoma. Cancer Discov (2017) 7(6):630–51. doi: 10.1158/2159-8290.CD-16-1022 28320776

[10] Kaczkowski B Tanaka Y Kawaji H Sandelin A Andersson R Itoh M. Transcriptome Analysis of Recurrently Deregulated Genes Across Multiple Cancers Identifies New Pan-Cancer Biomarkers. Cancer Res (2016) 76(2):216–26. doi: 10.1158/0008-5472.CAN-15-0484 26552699

[11] Demircioğlu D Cukuroglu E Kindermans M Nandi T Calabrese C Fonseca NA. A Pan-Cancer Transcriptome Analysis Reveals Pervasive Regulation Through Alternative Promoters. Cell (2019) 178(6):1465–77.e17. doi: 10.1016/j.cell.2019.08.018 31491388

[12] Huang KK Huang J Wu JKL Lee M Tay ST Kumar V. Long-Read Transcriptome Sequencing Reveals Abundant Promoter Diversity in Distinct Molecular Subtypes of Gastric Cancer. Genome Biol (2021) 22(1):44. doi: 10.1186/s13059-021-02261-x 33482911 PMC7821541

[13] Liu S. Increasing Alternative Promoter Repertories is Positively Associated With Differential Expression and Disease Susceptibility. PloS One (2010) 5(3):e9482. doi: 10.1371/journal.pone.0009482 20208995 PMC2830428

[14] Wilting RH Dannenberg JH. Epigenetic Mechanisms in Tumorigenesis, Tumor Cell Heterogeneity and Drug Resistance. Drug Resist Updates Rev Commentaries Antimicrob Anticancer Chemother (2012) 15(1-2):21–38. doi: 10.1016/j.drup.2012.01.008 22356866

[15] Nishiyama A Nakanishi M. Navigating the DNA Methylation Landscape of Cancer. Trends Genet (2021) 37(11):1012–27. doi: 10.1016/j.tig.2021.05.002 34120771

[16] Raval A Tanner SM Byrd JC Angerman EB Perko JD Chen SS. Downregulation of Death-Associated Protein Kinase 1 (DAPK1) in Chronic Lymphocytic Leukemia. Cell (2007) 129(5):879–90. doi: 10.1016/j.cell.2007.03.043 PMC4647864 17540169

[17] Magdinier F Wolffe AP. Selective Association of the Methyl-CpG Binding Protein MBD2 With the Silent P14/P16 Locus in Human Neoplasia. Proc Natl Acad Sci U States America (2001) 98(9):4990–5. doi: 10.1073/pnas.101617298 PMC33151 11309512

[18] Stirzaker C Song JZ Ng W Du Q Armstrong NJ Locke WJ. Methyl-CpG-Binding Protein MBD2 Plays a Key Role in Maintenance and Spread of DNA Methylation at CpG Islands and Shores in Cancer. Oncogene (2017) 36(10):1328–38. doi: 10.1038/onc.2016.297 27593931

[19] Zhao SG Chen WS Li H Foye A Zhang M Sjöström M. The DNA Methylation Landscape of Advanced Prostate Cancer. Nat Genet (2020) 52(8):778–89. doi: 10.1038/s41588-020-0648-8 PMC7454228 32661416

[20] Eden A Gaudet F Waghmare A Jaenisch R. Chromosomal Instability and Tumors Promoted by DNA Hypomethylation. Sci (New York NY) (2003) 300(5618):455. doi: 10.1126/science.1083557 12702868

[21] He N Park K Zhang Y Huang J Lu S Wang L. Epigenetic Inhibition of Nuclear Receptor Small Heterodimer Partner is Associated With and Regulates Hepatocellular Carcinoma Growth. Gastroenterology (2008) 134(3):793–802. doi: 10.1053/j.gastro.2008.01.006 18325392

[22] Zheng Y Huang Q Ding Z Liu T Xue C Sang X. Genome-Wide DNA Methylation Analysis Identifies Candidate Epigenetic Markers and Drivers of Hepatocellular Carcinoma. Briefings Bioinf (2018) 19(1):101–8. doi: 10.1093/bib/bbw094 27760737

[23] McCabe MT Brandes JC Vertino PM. Cancer DNA Methylation: Molecular Mechanisms and Clinical Implications. Clin Cancer Res (2009) 15(12):3927–37. doi: 10.1158/1078-0432.CCR-08-2784 PMC2715155 19509173

[24] Zhang YJ Chen Y Ahsan H Lunn RM Chen SY Lee PH. Silencing of Glutathione S-Transferase P1 by Promoter Hypermethylation and its Relationship to Environmental Chemical Carcinogens in Hepatocellular Carcinoma. Cancer Lett (2005) 221(2):135–43. doi: 10.1016/j.canlet.2004.08.028 15808399

[25] Xu RH Wei W Krawczyk M Wang W Luo H Flagg K. Circulating Tumour DNA Methylation Markers for Diagnosis and Prognosis of Hepatocellular Carcinoma. Nat Mater (2017) 16(11):1155–61. doi: 10.1038/nmat4997 29035356

[26] Yang JD Liu MC Kisiel JB. Circulating Tumor DNA and Hepatocellular Carcinoma. Semin Liver Dis (2019) 39(4):452–62. doi: 10.1055/s-0039-1688503 PMC10962397 31226727

[27] Cai J Chen L Zhang Z Zhang X Lu X Liu W. Genome-Wide Mapping of 5-Hydroxymethylcytosines in Circulating Cell-Free DNA as a non-Invasive Approach for Early Detection of Hepatocellular Carcinoma. Gut (2019) 68(12):2195–205. doi: 10.1136/gutjnl-2019-318882 PMC6872444 31358576

[28] Yang Y Chen L Gu J Zhang H Yuan J Lian Q. Recurrently Deregulated lncRNAs in Hepatocellular Carcinoma. Nat Commun (2017) 8:14421. doi: 10.1038/ncomms14421 28194035 PMC5316832

[29] Gao F Liang H Lu H Wang J Xia M Yuan Z. Global Analysis of DNA Methylation in Hepatocellular Carcinoma by a Liquid Hybridization Capture-Based Bisulfite Sequencing Approach. Clin Epigenet (2015) 7:86. doi: 10.1186/s13148-015-0121-1 PMC4546208 26300991

[30] Jin Y Lee WY Toh ST Tennakoon C Toh HC Chow PK. Comprehensive Analysis of Transcriptome Profiles in Hepatocellular Carcinoma. J Trans Med (2019) 17(1):273. doi: 10.1186/s12967-019-2025-x PMC6701074 31429776

[31] Li X Liu Y Salz T Hansen KD Feinberg A. Whole-Genome Analysis of the Methylome and Hydroxymethylome in Normal and Malignant Lung and Liver. Genome Res (2016) 26(12):1730–41. doi: 10.1101/gr.211854.116 PMC5131824 27737935

[32] Goldman MJ Craft B Hastie M Repečka K McDade F Kamath A. Visualizing and Interpreting Cancer Genomics Data *via* the Xena Platform. Nat Biotechnol (2020) 38(6):675–8. doi: 10.1038/s41587-020-0546-8 PMC7386072 32444850

[33] Liu Y Sun J Zhao M. ONGene: A Literature-Based Database for Human Oncogenes. J Genet Genomics (2017) 44(2):119–21. doi: 10.1016/j.jgg.2016.12.004 28162959

[34] Zhao M Kim P Mitra R Zhao J Zhao Z. TSGene 2.0: An Updated Literature-Based Knowledgebase for Tumor Suppressor Genes. Nucleic Acids Res (2016) 44(D1):D1023–D31. doi: 10.1093/nar/gkv1268 PMC4702895 26590405

[35] Chen S Zhou Y Chen Y Gu J. Fastp: An Ultra-Fast All-in-One FASTQ Preprocessor. Bioinf (Oxford England) (2018) 34(17):i884–i90. doi: 10.1093/bioinformatics/bty560 PMC6129281 30423086

[36] Dobin A Davis CA Schlesinger F Drenkow J Zaleski C Jha S. STAR: Ultrafast Universal RNA-Seq Aligner. Bioinf (Oxford England) (2013) 29(1):15–21. doi: 10.1093/bioinformatics/bts635 PMC3530905 23104886

[37] Li B Dewey CN. RSEM: Accurate Transcript Quantification From RNA-Seq Data With or Without a Reference Genome. BMC Bioinf (2011) 12:323. doi: 10.1186/1471-2105-12-323 PMC3163565 21816040

[38] Quinlan AR Hall IM. BEDTools: A Flexible Suite of Utilities for Comparing Genomic Features. Bioinf (Oxford England) (2010) 26(6):841–2. doi: 10.1093/bioinformatics/btq033 PMC2832824 20110278

[39] Xi Y Li W. BSMAP: Whole Genome Bisulfite Sequence MAPping Program. BMC Bioinf (2009) 10:232. doi: 10.1186/1471-2105-10-232 PMC2724425 19635165

[40] Sun D Xi Y Rodriguez B Park HJ Tong P Meong M. MOABS: Model Based Analysis of Bisulfite Sequencing Data. Genome Biol (2014) 15(2):R38. doi: 10.1186/gb-2014-15-2-r38 24565500 PMC4054608

[41] Zhou Y Zhou B Pache L Chang M Khodabakhshi AH Tanaseichuk O. Metascape Provides a Biologist-Oriented Resource for the Analysis of Systems-Level Datasets. Nat Commun (2019) 10(1):1523. doi: 10.1038/s41467-019-09234-6 30944313 PMC6447622

[42] Comoglio PM Trusolino L Boccaccio C. Known and Novel Roles of the MET Oncogene in Cancer: A Coherent Approach to Targeted Therapy. Nat Rev Cancer (2018) 18(6):341–58. doi: 10.1038/s41568-018-0002-y 29674709

[43] Chakraborty PK Mustafi SB Xiong X Dwivedi SKD Nesin V Saha S. MICU1 Drives Glycolysis and Chemoresistance in Ovarian Cancer. Nat Commun (2017) 8:14634. doi: 10.1038/ncomms14634 28530221 PMC5477507

[44] Luteijn RD Zaver SA Gowen BG Wyman SK Garelis NE Onia L. SLC19A1 Transports Immunoreactive Cyclic Dinucleotides. Nature (2019) 573(7774):434–8. doi: 10.1038/s41586-019-1553-0 PMC6785039 31511694

[45] Lev Maor G Yearim A Ast G. The Alternative Role of DNA Methylation in Splicing Regulation. Trends Genet (2015) 31(5):274–80. doi: 10.1016/j.tig.2015.03.002 25837375

[46] Zhao C Tao T Yang L Qin Q Wang Y Liu H. Loss of PDZK1 Expression Activates PI3K/AKT Signaling *via* PTEN Phosphorylation in Gastric Cancer. Cancer Lett (2019) 453:107–21. doi: 10.1016/j.canlet.2019.03.043 30930234

[47] Tao T Yang X Zheng J Feng D Qin Q Shi X. PDZK1 Inhibits the Development and Progression of Renal Cell Carcinoma by Suppression of SHP-1 Phosphorylation. Oncogene (2017) 36(44):6119–31. doi: 10.1038/onc.2017.199 28692056

[48] Ghosh MG Thompson DA Weigel RJ. PDZK1 and GREB1 are Estrogen-Regulated Genes Expressed in Hormone-Responsive Breast Cancer. Cancer Res (2000) 60(22):6367–75. 11103799

[49] Curradi M Izzo A Badaracco G Landsberger N. Molecular Mechanisms of Gene Silencing Mediated by DNA Methylation. Mol Cell Biol (2002) 22(9):3157–73. doi: 10.1128/MCB.22.9.3157-3173.2002 PMC133775 11940673

[50] Nabilsi NH Broaddus RR Loose DS. DNA Methylation Inhibits P53-Mediated Survivin Repression. Oncogene (2009) 28(19):2046–50. doi: 10.1038/onc.2009.62 19363521

[51] Tanenbaum DG Hall WA Colbert LE Bastien AJ Brat DJ Kong J. TNFRSF10C Copy Number Variation is Associated With Metastatic Colorectal Cancer. J Gastrointest Oncol (2016) 7(3):306–14. doi: 10.21037/jgo.2015.11.04 PMC4880759 27284460

[52] Zhou C Pan R Hu H Li B Dai J Ying X. TNFRSF10C Methylation is a New Epigenetic Biomarker for Colorectal Cancer. PeerJ (2018) 6:e5336. doi: 10.7717/peerj.5336 30225159 PMC6139245

[53] Tatenhorst L Senner V Püttmann S Paulus W. Regulators of G-Protein Signaling 3 and 4 (RGS3, RGS4) are Associated With Glioma Cell Motility. J Neuropathol Exp Neurol (2004) 63(3):210–22. doi: 10.1093/jnen/63.3.210 15055445

[54] Li W Si X Yang J Zhang J Yu K Cao Y. Regulator of G-Protein Signalling 3 and its Regulator microRNA-133a Mediate Cell Proliferation in Gastric Cancer. Arab J Gastroenterol Off Publ Pan-Arab Assoc Gastroenterol (2020) 21(4):237–45. doi: 10.1016/j.ajg.2020.07.011 32928707

[55] Zhang YJ Ahsan H Chen Y Lunn RM Wang LY Chen SY. High Frequency of Promoter Hypermethylation of RASSF1A and P16 and its Relationship to Aflatoxin B1-DNA Adduct Levels in Human Hepatocellular Carcinoma. Mol Carcinog (2002) 35(2):85–92. doi: 10.1002/mc.10076 12325038

[56] Grawenda AM O'Neill E. Clinical Utility of RASSF1A Methylation in Human Malignancies. Br J Cancer (2015) 113(3):372–81. doi: 10.1038/bjc.2015.221 PMC4522630 26158424

[57] Pefani DE Pankova D Abraham AG Grawenda AM Vlahov N Scrace S. TGF-β Targets the Hippo Pathway Scaffold RASSF1A to Facilitate YAP/SMAD2 Nuclear Translocation. Mol Cell (2016) 63(1):156–66. doi: 10.1016/j.molcel.2016.05.012 27292796

[58] Yu J Ni M Xu J Zhang H Gao B Gu J. Methylation Profiling of Twenty Promoter-CpG Islands of Genes Which may Contribute to Hepatocellular Carcinogenesis. BMC Cancer (2002) 2:29. doi: 10.1186/1471-2407-2-29 12433278 PMC139988

[59] Xiang J Qiu W Wang X Zhou F Wang Z Liu S. Efficient Downregulation of ErbB-2 Induces TACC1 Upregulation in Breast Cancer Cell Lines. Oncol Rep (2013) 29(4):1517–23. doi: 10.3892/or.2013.2253 23354013

[60] Ding A Zhao W Shi X Yao R Zhou F Yue L. Impact of NPM, TFF3 and TACC1 on the Prognosis of Patients With Primary Gastric Cancer. PloS One (2013) 8(12):e82136. doi: 10.1371/journal.pone.0082136 24358147 PMC3864846

[61] Lauffart B Vaughan MM Eddy R Chervinsky D DiCioccio RA Black JD. Aberrations of TACC1 and TACC3 are Associated With Ovarian Cancer. BMC Women's Health (2005) 5:8. doi: 10.1186/1472-6874-5-8 15918899 PMC1175095

[62] Qu F Lorenzo DN King SJ Brooks R Bear JE Bennett V. Ankyrin-B is a PI3P Effector That Promotes Polarized α5β1-Integrin Recycling *via* Recruiting RabGAP1L to Early Endosomes. eLife (2016) 5:e20417. doi: 10.7554/eLife.20417 27718357 PMC5089861

[63] Lou S Gao H Hong H Zhu Z Zhao H. Inhibition of Retinoic Acid Receptor α Phosphorylation Represses the Progression of Triple-Negative Breast Cancer *via* Transactivating miR-3074-5p to Target DHRS3. J Exp Clin Cancer Res (2021) 40(1):141. doi: 10.1186/s13046-021-01941-7 33902658 PMC8074416

[64] de Thé H Lavau C Marchio A Chomienne C Degos L Dejean A. The PML-RAR Alpha Fusion mRNA Generated by the T (15,17) Translocation in Acute Promyelocytic Leukemia Encodes a Functionally Altered RAR. Cell (1991) 66(4):675–84. doi: 10.1016/0092-8674(91)90113-d 1652369

[65] Khetchoumian K Teletin M Tisserand J Mark M Herquel B Ignat M. Loss of Trim24 (Tif1alpha) Gene Function Confers Oncogenic Activity to Retinoic Acid Receptor Alpha. Nat Genet (2007) 39(12):1500–6. doi: 10.1038/ng.2007.15 18026104

[66] Sano K Takayama T Murakami K Saiki I Makuuchi M. Overexpression of Retinoic Acid Receptor Alpha in Hepatocellular Carcinoma. Clin Cancer Res (2003) 9(10 Pt 1):3679–83. 14506158

[67] Zhang L Shay JW. Multiple Roles of APC and its Therapeutic Implications in Colorectal Cancer. J Natl Cancer Inst (2017) 109(8):djw332. doi: 10.1093/jnci/djw332 28423402 PMC5963831

[68] Jung Y-S Stratton SA Lee SH Kim M-J Jun S Zhang J. TMEM9-V-ATPase Activates Wnt/β-Catenin Signaling *Via* APC Lysosomal Degradation for Liver Regeneration and Tumorigenesis. Hepatology (2021) 73(2):776–94. doi: 10.1002/hep.31305 PMC7647947 32380568

[69] Kim H-S Vassilopoulos A Wang R-H Lahusen T Xiao Z Xu X. SIRT2 Maintains Genome Integrity and Suppresses Tumorigenesis Through Regulating APC/C Activity. Cancer Cell (2011) 20(4):487–99. doi: 10.1016/j.ccr.2011.09.004 PMC3199577 22014574

[70] Colnot S Decaens T Niwa-Kawakita M Godard C Hamard G Kahn A. Liver-Targeted Disruption of Apc in Mice Activates Beta-Catenin Signaling and Leads to Hepatocellular Carcinomas. Proc Natl Acad Sci USA (2004) 101(49):17216–21. doi: 10.1073/pnas.0404761101 PMC535370 15563600

[71] Handa O Goda K Handa Y Fukushima S Osawa M Murao T. PDZK1 Induces Resistance to Apoptosis in Esophageal Adenocarcinoma Cells. Esophagus (2021) 18(3):655–62. doi: 10.1007/s10388-021-00819-z 33586076

[72] Kim H Abd Elmageed ZY Davis C El-Bahrawy AH Naura AS Ekaidi I. Correlation Between PDZK1, Cdc37, Akt and Breast Cancer Malignancy: The Role of PDZK1 in Cell Growth Through Akt Stabilization by Increasing and Interacting With Cdc37. Mol Med (2014) 20:270–9. doi: 10.2119/molmed.2013.00166 PMC4107102 24869908

[73] Inoue J Otsuki T Hirasawa A Imoto I Matsuo Y Shimizu S. Overexpression of PDZK1 Within the 1q12-Q22 Amplicon is Likely to be Associated With Drug-Resistance Phenotype in Multiple Myeloma. Am J Pathol (2004) 165(1):71–81. doi: 10.1016/S0002-9440(10)63276-2 15215163 PMC1618545

[74] Chen T Chen X Zhang S Zhu J Tang B Wang A. The Genome Sequence Archive Family: Toward Explosive Data Growth and Diverse Data Types. Genomics Proteomics Bioinf (2021) S1672-0229(21):00163–7. doi: 10.1016/j.gpb.2021.08.001 PMC9039563 34400360

[75] Database Resources of the National Genomics Data Center. China National Center for Bioinformation in 2021. Nucleic Acids Res (2021) 49(D1):D18–d28. doi: 10.1093/nar/gkaa1022 33175170 PMC7779035

