# Corrigendum: A genome-wide investigation of effects of aberrant DNA methylation on the usage of alternative promoters in hepatocellular carcinoma

**DOI:** 10.3389/fonc.2023.1253552

**Published:** 2023-08-31

**Authors:** Yuting Dong, Xiaozhao Liu, Bijun Jiang, Siting Wei, Bangde Xiang, Ruichu Liao, Qiuyan Wang, Ximiao He

**Affiliations:** ^1^ Department of Physiology, School of Basic Medical Science, Huazhong University of Science and Technology, Wuhan, China; ^2^ Center for Genomics and Proteomics Research, School of Basic Medicine, Tongji Medical College, Huazhong University of Science and Technology, Wuhan, China; ^3^ Hubei Key Laboratory of Drug Target Research and Pharmacodynamic Evaluation, Huazhong University of Science and Technology, Wuhan, China; ^4^ Department of Hepatobiliary Surgery, Guangxi Medical University Cancer Hospital, Nanning, China; ^5^ Center for Genomic and Personalized Medicine, Guangxi Medical University, Nanning, China; ^6^ Guangxi Key Laboratory for Genomic and Personalized Medicine, Guangxi, Collaborative Innovation Center for Genomic and Personalized Medicine, Nanning, China

**Keywords:** hepatocellular carcinoma, alternative promoters, DNA methylation, diagnostic model, survival prediction

In the published article, there was an error in the legend for **Supplementary Figures 6M, N**. The sample correlation clustering was performed using “promoter activity” instead of “WGBS”. The correct material statement appears below.

(M) Heatmap shows the correlation of three pairs of promoter activity in GSE70091.

(N) Heatmap shows the better performance correlation of two pairs of promoter activity in GSE70091 after removing N3 and T3 pairs.

In the published article, there was a typographical error. The gene name “*RABGAP1L*” was mistakenly written as “*RABGAPL1*”.

A correction has been made to **Results,** *The Methylation Regulated APs Could Be Used as Tumor Diagnostic Markers*, Paragraph 1. This sentence previously stated:

“The six mrAPs were clustered into four upregulated mrAPs (prmtr.53735 of *TNFRSF10*, prmtr.32651 of *RGS3*, prmtr.36049 of *CCDC150*, and prmtr.5237 of *RASSF1*) and two downregulated mrAPs (prmtr.37640 of *TACC1* and prmtr.39585 of *RABGAPL1*) by promoter activities (**Figure 4D**, upper; **Table 1**; **Table S5**).”

The corrected sentence appears below:

“The six mrAPs were clustered into four upregulated mrAPs (prmtr.53735 of *TNFRSF10*, prmtr.32651 of *RGS3*, prmtr.36049 of *CCDC150*, and prmtr.5237 of *RASSF1*) and two downregulated mrAPs (prmtr.37640 of *TACC1* and prmtr.39585 of *RABGAP1L*) by promoter activities (**Figure 4D**, upper; **Table 1**; **Table S5**).”

According to the guidelines of Ethics Statement, the authors replaced the previous statement “Written informed consent was obtained from the individual(s) for the publication of any potentially identifiable images or data included in this article” with the following statement.

“The studies involving humans were approved by the Ethics Committee of Guangxi Medical University Cancer Hospital. The studies were conducted in accordance with the local legislation and institutional requirements. The participants provided their written informed consent to participate in this study.”

The authors apologize for these errors and amendments and state that this does not change the scientific conclusions of the article in any way. The original article has been updated.

