# Supplementary figures and images for: A Genome-Wide Investigation of Effects of Aberrant DNA Methylation on the Usage of Alternative Promoters in Hepatocellular Carcinoma
Source: Front Oncol. 2022 Jan 17;11:780266. doi: 10.3389/fonc.2021.780266 (PMC8803206; doi:10.3389/fonc.2021.780266)

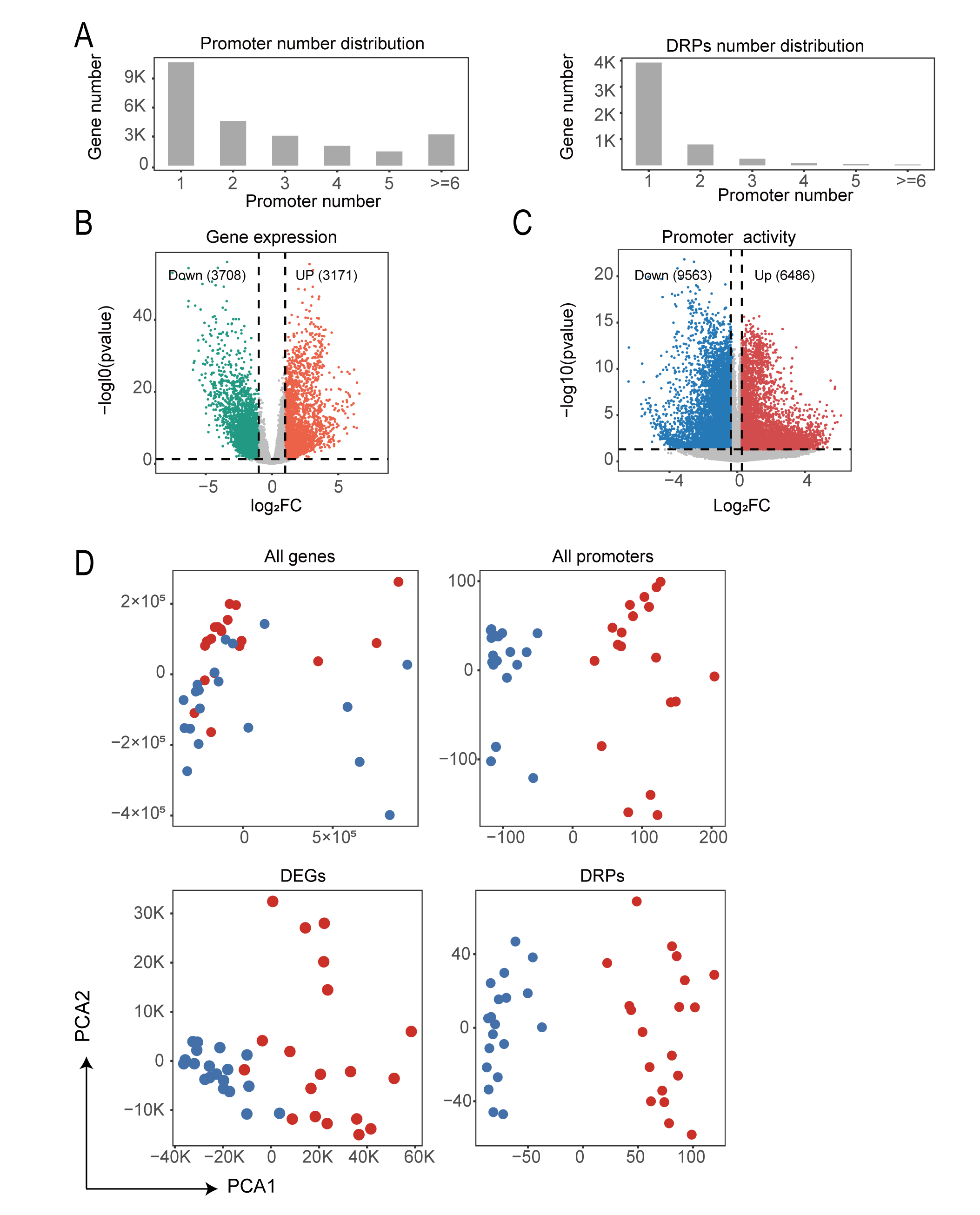

Supplement: Supplementary Figure 1 — Comparative analysis of promoter activity and gene expression in HCC. Related to Figure 1. (A) The number of promoters with activities in HCC per gene (left); number of differentially regulated promoters (DRPs) with activities in HCC per gene. (B–C) Volcano plot showing the log2 (fold change) in gene expression (B) and promoter activity (C) in HCC and adjust normal tissues. (D) Principal component analysis (PCA) clustering the sequenced samples by FPKM for all genes or DEGs and by promoter activities for all promoters or DRPs. Samples were colored by sample types (dark red: HCC; dark blue: adjacent normal tissue). [file Image_1.tif]

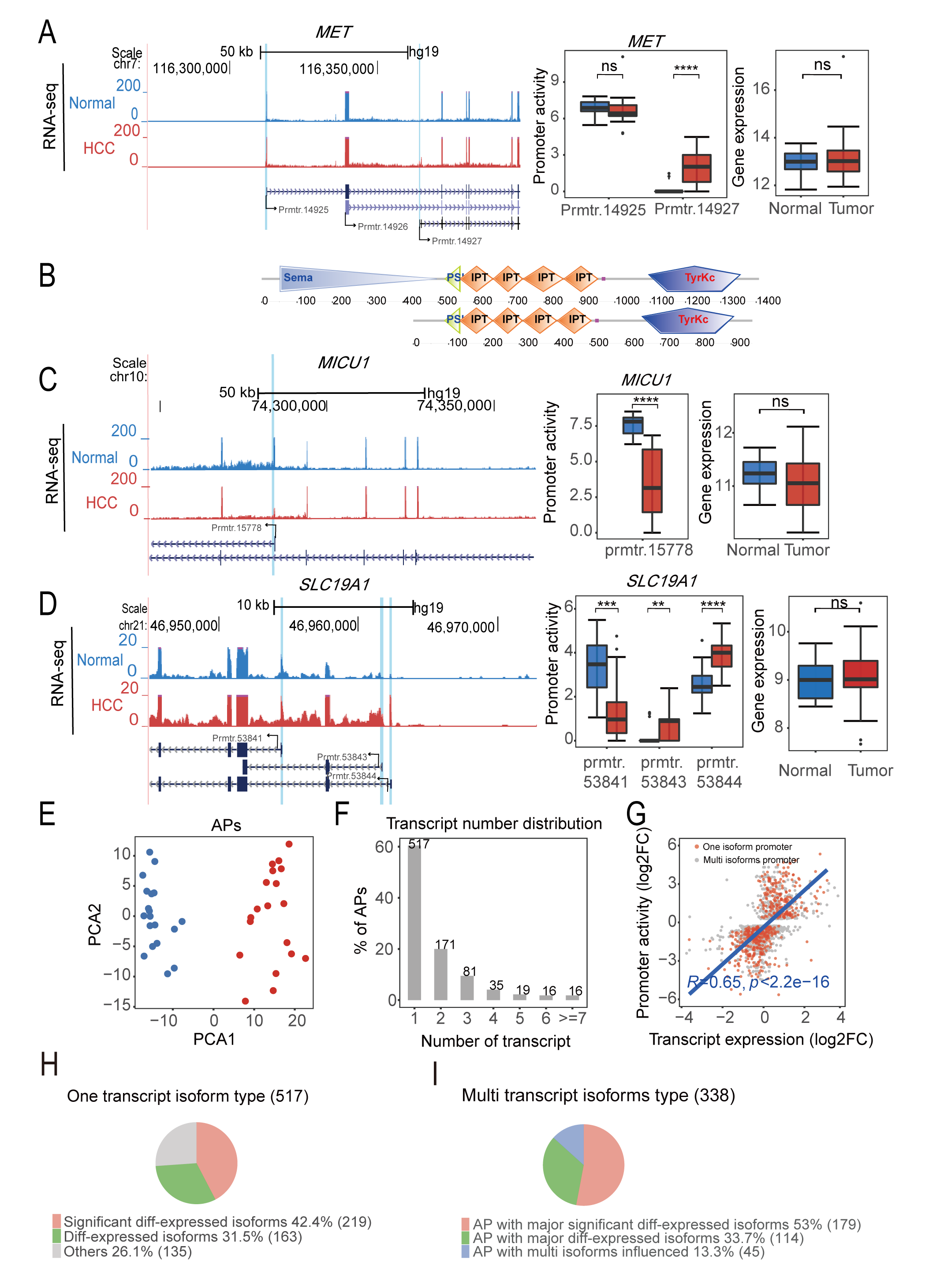

Supplement: Supplementary Figure 2 — Examples of alternative promoters (APs). Related to Figure 2. (A) UCSC genome browser screenshot showing mean read count of prmtr.14925 and prmtr.14927 at the MET gene locus in HCC (red track) and normal tissues (blue track). The boxplot showing the expression of gene RARA in tumor and normal was nearly the same. The boxplot showing promoter activity of prmtr.14927 was significantly higher in HCC samples. ****p-value <0.0001 (ANOVA, p-value = 4.57e-05). (B) The protein domains of isoforms initiated from prmtr.14925 and prmtr.14927. (C) UCSC genome browser screenshot showing mean read count of prmtr.15778 at the MICU1 gene locus in HCC (red track) and normal tissues (blue track). The boxplot showing the expression of gene MICU1 in tumor and normal was nearly the same. The boxplot showing promoter activity of prmtr.15778 was significantly lower in HCC samples. ****p-value <0.0001 (ANOVA, p-value = 1.34e-08). (D) UCSC genome browser screenshot showing mean read count of prmtr.53841, prmtr.53843, and prmtr.53844 at the SLC19A1 gene locus in HCC (red track) and normal tissues (blue track). The boxplot showing the expression of gene SLC19A1 in tumor and normal was nearly the same. The boxplot showing promoter activity of prmtr.53841 was significantly lower in HCC tissues, but prmtr.53843, and prmtr.53844 were significantly higher in HCC samples. ****p-value <0.0001 (ANOVA, prmtr.53841: p-value = 1.16e-04; prmtr.53843: p-value = 7.58e-03; prmtr.53844: p-value = 7.84e-06). (E) PCA plot showing normal (blue dots) and HCC (red dots) samples can be clustered by activities of all APs. (F) The number of transcripts with same TSSs in HCC per APs. (G) Scatter plots showing the correlation between transcript expression (log2FC) and promoter activity (log2FC) of APs. (H) The pie chart showing the percentage of significant differential expressed isoforms (cancer versus normal, p < 0.05), differential expressed isoforms (cancer versus normal, |fold-change| > 1.2) and others in APs wit [file Image_2.tif]

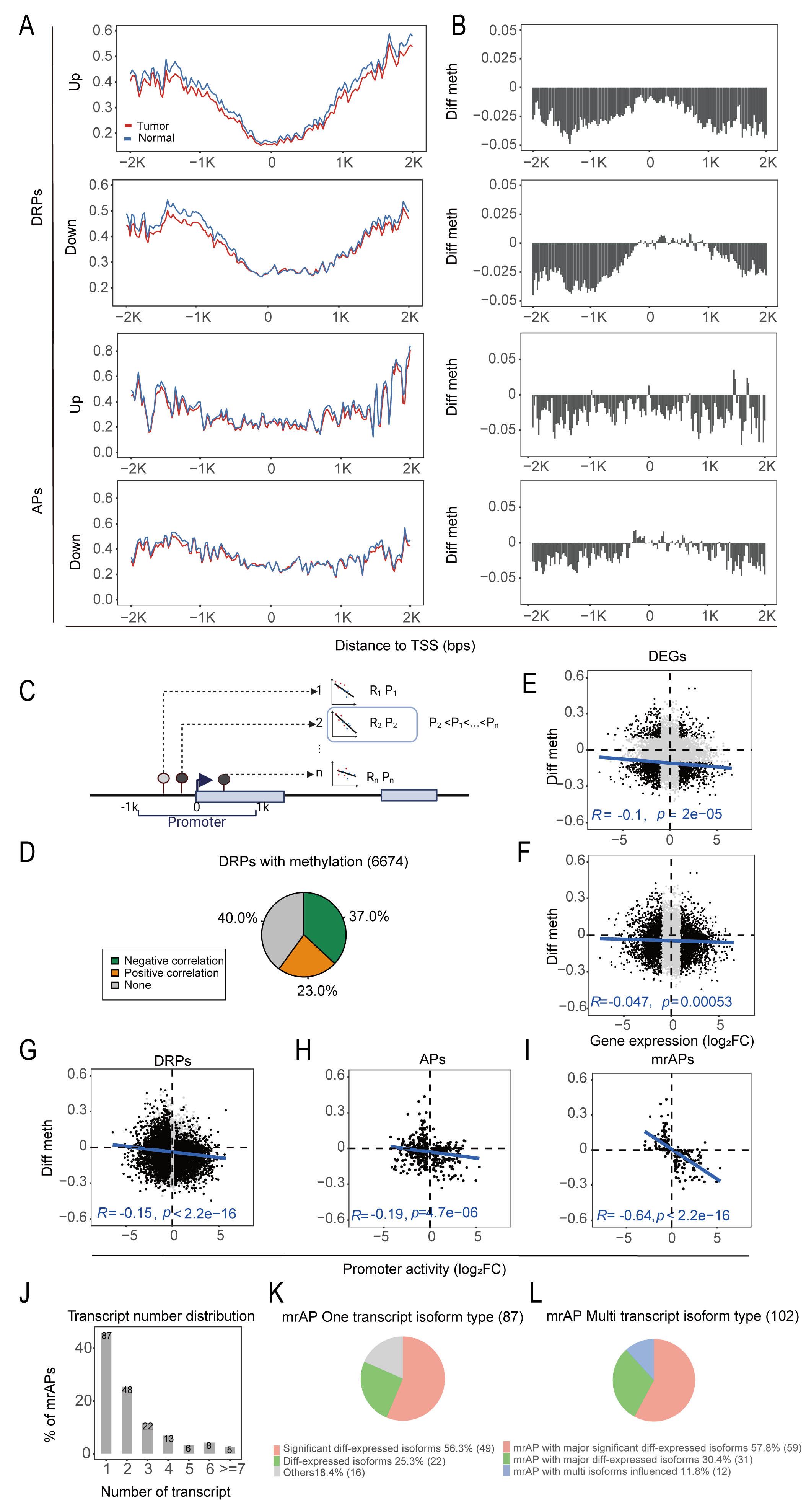

Supplement: Supplementary Figure 3 — Correlation analysis of promoter activity and methylation status. Related to Figure 3. (A) The methylation profile showing mean methylation levels of TSS nearby region (±2k) of the DRPs (Top) and APs (Bottom). Up-regulated and down-regulated promoters were shown separately. The blue line and red line represent normal and HCC samples respectively. Methylation profile was smoothed by 50 bps sliding windows with 25 bps steps. (B) The differential methylation (Tumor - Normal) of each window was calculated and plotted corresponding to (A) (C) The Schematic illustrates the method to screen the CpG sites with the minimal p-value correlation. Correlation tests between each CpG methylation status and the promoter activity were performed, and the CpG site with the minimal p-value was selected. (D) The proportions of correlation categories between the promoter activities of DRPs and their methylation status are shown in the pie chart. Negative correlation, positive correlation, and none correlation are colored by green, orange, and grey respectively. (E–F) Scatter plots showing the correlation between methylation (HCC – normal) and promoter activity by normalized change fold for DEGs. (G-I) Scatter plots showing the correlation between differential methylation (HCC – normal) and promoter activity by normalized change fold for DRPs (G), APs (H) and mrAPs (I). The representative CpG sites were filtered from the ±1k upstream and downstream of TSS (see also Methods). (J) The number of transcripts with same TSSs in HCC per mrAP. (K) The pie chart showing the percentage of significant differential expressed isoforms (cancer versus normal, p < 0.05), differential expressed isoforms (cancer versus normal, |fold-change| > 1.2) and others in mrAPs with only one transcript isoform. (L) The pie chart showing the percentage of mrAP with major differential expressed isoforms, mrAP with major diff isoform (contain one or more transcript isoform express different, |fold-change| > 1.2) and mul [file Image_3.tif]

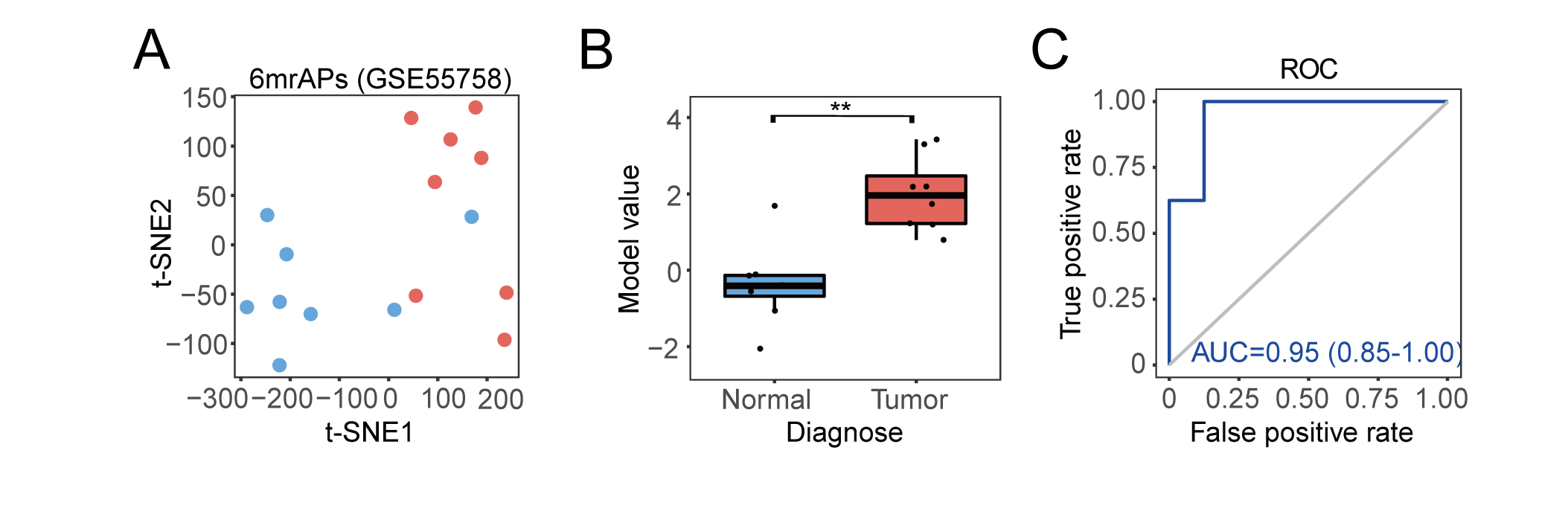

Supplement: Supplementary Figure 4 — Effectiveness of the diagnostic model based on 6 mrAPs in another independent test dataset. Related to Figure 4. (A) t-SNE plot showing normal (blue dots) and HCC (red dots) samples could also be grouped by the activities of six mrAPs in the independent test dataset of GSE55758. (F) Boxplot showing the significant different model scores of HCC and normal sample of the test dataset of GSE55758. **p-value <0.01 (Wilcoxon test, p-value = 0.0011) (G) ROC curve showing the performance and prediction accuracy of the diagnostic model in the test dataset of GSE55758. [file Image_4.tif]

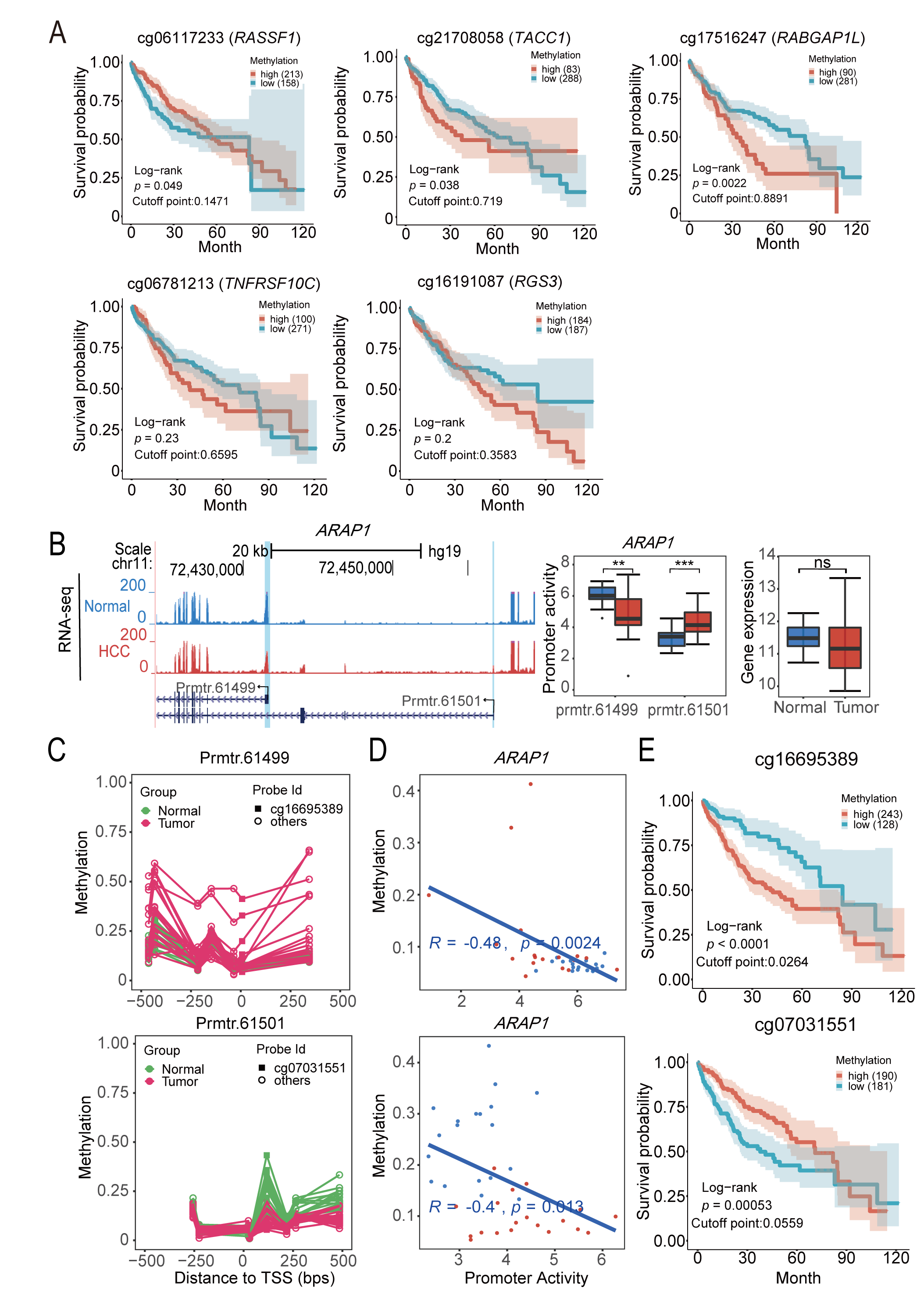

Supplement: Supplementary Figure 5 — The methylation status of the case with switch promoter activity usage predicts survival of HCC patients. Related to Figure 5. (A) The 10 years overall survival curve of methylation levels of cg06781213 (related to TNFRSF10C), cg16191087 (related to RGS3), cg06117233 (related to RASSF1), cg21708058 (related to TACC1), cg17516247 (related to RABGAP1L) in TCGA-LIHC patients in high and low methylation cohort. (B) UCSC genome browser screenshot showing mean read count (top 2 tracks) and 450 K methylation beta values (bottom 2 tracks) at the ARAP1 gene locus of HCC (red) and normal tissues (blue or green). The boxplot shows the gene expression in tumor and normal was roughly equal. The activity of prmtr.61499 is significantly lower in HCC tissues, but prmtr.61501 is significantly higher in HCC samples **p-value <0.01 (ANOVA, prmtr.61499: p-value = 1.48e-03; prmtr.61501: p-value = 3.24e-04). (C) Methylation beta value of ± 500 bps relative to TSS of prmtr.61499 (upper) and prmtr.61501 (bottom). Normal and tumor samples are colored by green and red dots, dots from the same sample were connected by lines. Cg16695389 and cg07031551 with the lowest p-value for the correlation test were marked and screened for calculation in (D) and (E). (D) The scatter plot showing the negative correlation between promoter activities of prmtr.61499 and methylation beta values of cg16695389 in HCC (red) and normal (blue) samples. A similar negative correlation exhibits between prmtr.61501 and methylation beta values of cg07031551. (E) The 10 years overall survival curve of methylation levels of cg16695389 and cg07031551 in TCGA-LIHC patients in high and low methylation cohort, showing both methylations of cg16695389 and cg07031551 was significantly associated with survival in HCC. [file Image_5.tif]

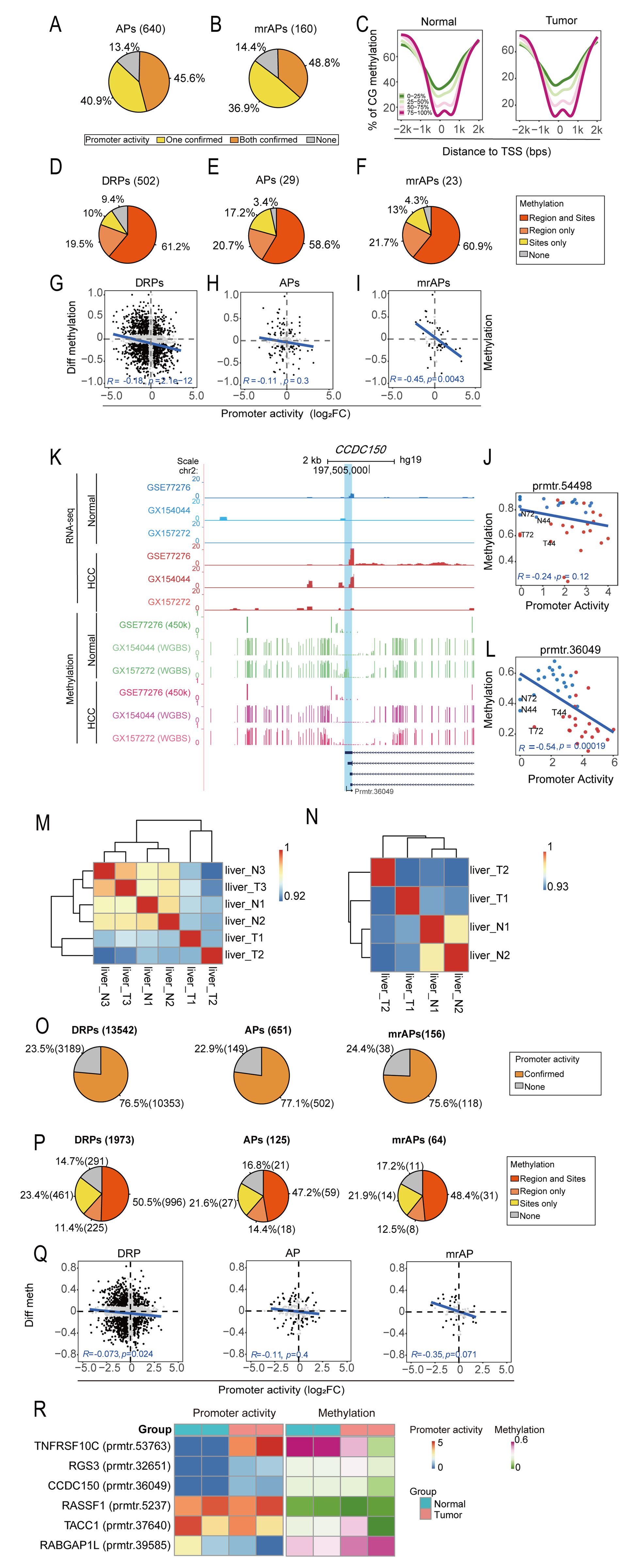

Supplement: Supplementary Figure 6 — Comprehensive verification using RNA-seq and WGBS in HCC patients. Related to Figure 6. (A–B) The pie chart shows in detail the percentage of all identified APs (A) and mrAPs (B) in GSE77276 with differential promoter activities being confirmed by our validation dataset of RNA-seq. (C) Methylation levels of CpGs within ± 2kbps upstream and downstream relative to TSS were assessed in four groups classified by the quartiles of promoter activities. Green to red represents the promoter activities levels from 0 to 100%. Methylation profile was smoothed by gam (Generalized Additive Models). (D-F) The pie chart showing the percentage of all identified DRPs (D), APs (E) and mrAPs (F) in GSE77276 with differential methylation status being confirmed by our validation dataset of WGBS. Changes of methylation of CpG site (site) with over 0.2 in both public 450K data and our validation WGBS data, and also with the same alteration trends, would be regarded as confirmed. The promoter region (region) mean methylation were adopted for testing if the methylation of a CpG site is not available in WGBS dataset. (G-I) Scatter plots showing the correlation between differential methylation (HCC – normal) and promoter activity by normalized change fold for DRPs (G), APs (H) and mrAPs (I) The available above selected CpG methylation value was used to calculate delta methylation. Only these black dots were used for the Pearson correlation test. (J) The scatter plot shows the correlation test result by GSE77276 and validation RNA-seq dataset between the activity of PDZK1 (prmtr.54498) and methylation levels of its selected CpG (cg19353949) methylation or region mean methylation value, normal and tumor samples are colored by blue and red, validation samples are marked in the plot. (K) UCSC genome browser screenshot showing the promoter activities and methylation of gene CCDC150 in both public dataset of GSE77276 and our validation datasets. GSE77276 RNA-seq tracks represent mean read counts of [file Image_6.tif]
